# Single-cell disulfidptosis regulator patterns guide intercellular communication of tumor microenvironment that contribute to kidney renal clear cell carcinoma progression and immunotherapy

**DOI:** 10.3389/fimmu.2024.1288240

**Published:** 2024-01-16

**Authors:** Kangjie Xu, Dongling Li, Jinke Qian, Yanhua Zhang, Minglei Zhang, Hai Zhou, Xuefeng Hou, Jian Jiang, Zihang Zhang, Hang Sun, Guodong Shi, Hua Dai, Hui Liu

**Affiliations:** ^1^ Central Laboratory Department, Binhai County People’s Hospital, Yancheng, Jiangsu, China; ^2^ Nephrology Department, Binhai County People’s Hospital, Yancheng, Jiangsu, China; ^3^ Urology Department, Binhai County People’s Hospital, Yancheng, Jiangsu, China; ^4^ Obstetrics and Gynecology Department, Binhai County People’s Hospital, Yancheng, Jiangsu, China; ^5^ Oncology Department, Binhai County People’s Hospital, Yancheng, Jiangsu, China; ^6^ Pathology Department, Binhai County People’s Hospital, Yancheng, Jiangsu, China; ^7^ Medical Department, Binhai County People’s Hospital, Yancheng, Jiangsu, China; ^8^ Yangzhou University Clinical Medical College, Jiangsu Key Laboratory of Experimental & Translational Non-coding RNA Research, Yancheng, Jiangsu, China

**Keywords:** kidney renal clear cell carcinoma, single-cell, disulfidptosis, tumor microenvironment, prognosis, immunotherapy, multiple immunohistochemistry

## Abstract

**Background:**

Disulfidptosis, an emerging type of programmed cell death, plays a pivotal role in various cancer types, notably impacting the progression of kidney renal clear cell carcinoma (KIRC) through the tumor microenvironment (TME). However, the specific involvement of disulfidptosis within the TME remains elusive.

**Methods:**

Analyzing 41,784 single cells obtained from seven samples of KIRC through single-cell RNA sequencing (scRNA-seq), this study employed nonnegative matrix factorization (NMF) to assess 24 disulfidptosis regulators. Pseudotime analysis, intercellular communication mapping, determination of transcription factor activities (TFs), and metabolic profiling of the TME subgroup in KIRC were conducted using Monocle, CellChat, SCENIC, and scMetabolism. Additionally, public cohorts were utilized to predict prognosis and immune responses within the TME subgroup of KIRC.

**Results:**

Through NMF clustering and differential expression marker genes, fibroblasts, macrophages, monocytes, T cells, and B cells were categorized into four to six distinct subgroups. Furthermore, this investigation revealed the correlation between disulfidptosis regulatory factors and the biological traits, as well as the pseudotime trajectories of TME subgroups. Notably, disulfidptosis-mediated TME subgroups (DSTN+CD4T-C1 and FLNA+CD4T-C2) demonstrated significant prognostic value and immune responses in patients with KIRC. Multiple immunohistochemistry (mIHC) assays identified marker expression within both cell clusters. Moreover, CellChat analysis unveiled diverse and extensive interactions between disulfidptosis-mediated TME subgroups and tumor epithelial cells, highlighting the TNFSF12-TNFRSF12A ligand-receptor pair as mediators between DSTN+CD4T-C1, FLNA+CD4T-C2, and epithelial cells.

**Conclusion:**

Our study sheds light on the role of disulfidptosis-mediated intercellular communication in regulating the biological characteristics of the TME. These findings offer valuable insights for patients with KIRC, potentially guiding personalized immunotherapy approaches.

## Introduction

According to Cancer Statistics for, 2023, kidney cancer stands as the second most prevalent tumor within the urinary system, demonstrating an annual growth rate of approximately 1%. Estimates for the year, 2023 project diagnoses of 52,360 cases among males and 29,440 among females in the United States, accounting for nearly half of all urinary system cancers. Additionally, it is anticipated that 14,890 patients will succumb to kidney cancer (1). Within the various histological subtypes of renal cell carcinoma (RCC), kidney renal clear cell carcinoma (KIRC) stands out as the most prevalent, constituting approximately 75%-80% of all RCC cases (2). KIRC tumors exhibit heterogeneity, high metastatic potential, and a poor prognosis (3). Therefore, comprehending the molecular mechanisms underlying KIRC is crucial, holding the potential to significantly enhance survival outcomes for patients.

Programmed cell death (PCD) encompasses metabolic, structural, and functional cellular disorders that result in irreversible damage. It is associated with cellular homeostasis, tissue remodeling, and tumor development (4). Within KIRC, various PCD forms have been identified, including anoikis (5), pyroptosis (6), necroptosis (7), and cuproptosis (8). Disulfidptosis, a recently discovered form of PCD, is characterized by inadequate cellular uptake of cysteine and NADPH supply. Upon NADPH depletion, abnormal disulfide bonds accumulate in the cell cytoskeleton, leading to actin filament disruption and eventual collapse of the actin cytoskeleton, culminating in cell death (9). Actin, a multifunctional cytoskeletal protein involved in cellular morphology, maintenance, differentiation, and intracellular transport, is also linked to disulfide formation (10, 11). Alterations in the cytoskeleton of animal and plant cells may positively influence the initiation and regulation of PCD (12, 13). Crucially, disulfidptosis-related genes play vital roles in tumor growth, development, invasion, and are closely associated with KIRC’s pathogenesis and prognosis (14, 15).

The tumor microenvironment (TME) significantly influences KIRC tumor progression and metastasis (16, 17). Mass cytometry analysis revealed extensive infiltration of CD8^+^ PD-1^+^ T cells within KIRC tumors, indicating heightened lymphocyte presence in the TME (18). Immunotherapy targeting the TME, primarily employing immune checkpoint inhibitors (ICIs) like anti-PD-1/PD-L1 and CTLA-4 inhibitors (19, 20), has revolutionized cancer treatment. In KIRC, T cell exhaustion is strongly linked to poor prognosis, potentially contributing to the immunosuppressive nature of the TME (21). In addition, the presence of tumor-infiltrating lymphocytes (TILs) is associated with a favorable prognosis in ccRCC. ccRCC also contains a significant number of myeloid-derived suppressor cells (MDSCs), which possess the ability to hinder tumor immune responses (22). Single-cell transcriptomics, unlike traditional bulk analysis, can uncover intercellular communication among different TME cell subtypes, including CAFs, tumor-associated macrophages (TAMs), B cells, and T cells (23, 24). Aleksandar et al. identified upregulated tumor-specific macrophage subsets involving TREM2/APOE/C1Q as potential prognostic biomarkers and therapeutic targets for KIRC recurrence (25). Recently, Songyun et al. revealed a strong correlation between disulfidptosis-related genes and tumor-infiltrating immune cells (TIICs), particularly macrophages linked to SLC7A11 and SLC3A2 (26). However, there is limited research on specific disulfidptosis-mediated TME subgroups and their effect on patient prognosis and intercellular communication.

This study investigated the effect of disulfidptosis on major TME components—epithelial cells, fibroblasts, myeloids, B cells, and T cells (27)—utilizing single-cell RNA-seq (scRNA-seq) data from 7 KIRC tumor samples comprising 41,784 cells. Applying nonnegative matrix factorization (NMF) clustering to major KIRC subgroups using disulfidptosis regulatory factors, this study investigated the intricate interplay between these disulfidptosis-mediated TME subgroups and tumor epithelial cells. This study aimed to explore signaling pathways, functional enrichments, transcriptional features, immune characteristics, metabolic pathways, and prognostic implications within these distinct subgroups. Although TME has been shown to play an important role in tumor progression, the signaling molecules involved in intercellular communication in TME are poorly understood. Consequently, this study sheds light on the potential role of disulfidptosis in governing intercellular communication among various TME subgroups and tumor cells, thereby influencing KIRC progression.

## Materials and methods

### Data source

To explore the intratumor heterogeneity of KIRC, tumor scRNA-seq data were gathered from 7 patients with KIRC, aiming to scrutinize the landscape of 24 disulfidptosis regulators. These regulators were pinpointed using a genome-wide CRISPR-Cas9 screen and proteomic analyses conducted by Liu et al. (9) on SLC7A11high cells. The complete dataset, comprising a total of 41,784 single-cell scRNA-seq gene expression matrices from tumor samples of 7 patients, was acquired from the Gene Expression Omnibus (GEO) dataset (GSE210038) (www.ncbi.nlm.nih.gov/geo). Additionally, bulk RNA-seq data from 646 patients with KIRC were sourced from The Cancer Genome Atlas (TCGA) database (https://portal.gdc.cancer.gov/) and the GEO dataset (GSE29609). All data utilized or produced in this study are publicly accessible through previous publications or in the public domain.

### Visualization of TME cell types and subtypes in KIRC

Using the “Seurat” package within R software, Seurat objects were individually created based on scRNA-seq data from the 7 patients utilizing the CreateSeuratObject function. Subsequently, these objects were merged using the merge function to construct a comprehensive scRNA-seq gene expression matrix. Cells of subpar quality, including those containing less than 200 genes/cell, more than, 4000 genes/cell, or more than 15% mitochondrial genes, were filtered out. The scRNA-seq data underwent normalization using the NormalizeData function. Moreover, the FindVariableFeatures function was applied to identify the top, 2000 genes for data normalization. Additionally, the principal components (PCs) were computed based on the Seurat objects using the ScaleData and RunPCA functions. For this study, PC=12 and resolution=1.2 were chosen. The “t-SNE (t-distributed stochastic neighbor embedding)” algorithm was utilized to condense the topPCs for dimensionality reduction. Finally, the Idents function was employed to label the cells according to the TME cell types or subtypes, and the DimPlot function was used for visualization.

### Pseudotime trajectory analysis of disulfidptosis regulators for TME cells

To delve into the relationship between cell pseudotime trajectories and disulfidptosis regulators, the “Monocle” R package was used to analyze single-cell RNA data across all cell types in KIRC (28). Highly variable genes were selected based on the following thresholds: mean_expression ≥ 0.1 and dispersion_empirical ≥ 1 * dispersion_fit. The DDRTree method was employed for dimensionality reduction, with the parameter max_components set to 2. Subsequently, the function “plot_pseudotime_heatmap” was used to illustrate the pseudotime heatmap, showcasing temporal expression patterns of disulfidptosis regulators. Furthermore, the “plot_cell_trajectory” functions were utilized to visually represent pseudotime trajectories of diverse cell types within the KIRC TME. Thus, these analyses effectively demonstrate the dynamic regulation of disulfidptosis and its implications in the complex TME.

### NMF of disulfidptosis-mediated disulfidptosis regulators in TME cells

NMF unsupervised clustering represents a widely adopted technique in both data mining and machine learning. It functions by decomposing a data matrix into non-negative basis vectors and coefficient matrices (29). This method proves highly effective in unraveling the intricacies of the TME and identifying heterogeneity among tumor cells (30). To further investigate the influence of disulfidptosis-mediated regulator expression on various TME cell types, the “NMF” algorithm was employed along with the “snmf/r” method. This allowed for a comprehensive dimensionality reduction analysis involving 24 disulfidptosis regulatory factors across all TME cell types, enabling the identification of distinct cell subtypes within these groups. Noteworthy, these steps were performed using approaches that closely align with methodologies used previously (23, 31).

### Identification of the marker genes of disulfidptosis-related cell subtypes in TME

The FindAllMarkers function was utilized to determine the characteristic markers for each NMF cluster and discern different cell subtypes within these types. Genes were filtered based on a log-fold change threshold of 0.5 and a p-value below 0.01, with default parameters applied for the remaining settings. Additionally, employing the AddModuleScore function, module scores were computed using differentially expressed genes (DEGs) specific to each NMF cluster. The Dotplot visualization method was then utilized to represent the expression levels of top characteristic genes within each NMF cluster. Furthermore, the FeaturePlot was employed to illustrate the distribution of NMF cluster scores in the TME of KIRC. **Supplementary Table S1** provides detailed information on the specific gene sets used for comparing disulfidptosis-mediated TME subgroups.

### Functional enrichment analysis for NMF disulfidptosis-related subtypes

The “clusterProfiler” R package was utilized to identify marker genes within NMF clusters based on the Kyoto Encyclopedia of Genes and Genomes (KEGG) pathway database. Significance was determined by a corrected p.adjust < 0.05. Additionally, gene set variation analysis (GSVA) using 24 disulfidptosis regulatory factors was conducted to calculate enrichment scores for these NMF clusters. The “scMetabolism” R package, employing VISION methods, enables metabolic activity quantification at a single-cell resolution. This package comprises 85 KEGG pathways and 82 REACTOME pathways, facilitating comprehensive analysis (32). scMetabolism was employed to assess metabolic activity across different NMF clusters, and results were visualized using the DotPlot.metabolism function.

### SCENIC analysis for NMF disulfidptosis-related subtypes

SCENIC analysis was employed to investigate the gene regulatory network of transcription factors (TFs) in KIRC (33) using the aertslab database (https://resources.aertslab.org/). The database provided crucial files, namely, “*hg19-500bp-upstream-7species.mc9nr.feather*” which contained the genomic coordinates of the 500bp upstream regions of all human genes in the human genome based on the hg19 version, and “*hg19-tss-centered-10kb-7species.mc9nr.feather*” which encompassed the genomic coordinates of the 10 kb regions centered around the transcription start site (TSS) for the same genomic version. These files enabled TSS identification within KIRC scRNA-seq data, allowing recognition of potential TF-target relationships and construction of a co-expression gene network. Further analysis focused on TFs with a Benjamini-Hochberg false discovery rate corrected p-value < 0.05.

### Cell-cell communication analysis for NMF disulfidptosis-related subtypes

Utilizing the human and mouse ligand-receptor interaction database, the “CellChat” R package was employed to analyze intercellular communication networks in scRNA-seq data across various cell clusters (34). Initially, overexpressed genes in NMF clusters were identified, focusing on ligands or receptors, and their expression data were projected into a protein-protein interaction network. The collective intercellular communication network was computed using the computeCommunProbPathway and aggregateNet functions. Subsequently, the cell-cell communication network and visualized communication strength were analyzed using the netVisual_circle functions. Finally, leveraging the human ligand-receptor pairs database, the interactions between cell types and visualized ligand-receptor interactions’ strength across different cell clusters were examined using the netVisual_bubble function.

### Survival analyses with disulfidptosis-related signatures in RNA-seq

Bulk RNA-seq offers substantial clinical information. Thus, bulk RNA-seq data were integrated to explore the effect of disulfidptosis-related clusters on patient prognosis. To pinpoint distinct disulfidptosis-related genes for each NMF cluster, the FindAllmarker function was used, setting a logfc threshold at 0.5. Next, employing the GSVA method, gene scores for these identified genes were calculated from two publicly available KIRC datasets: TCGA-KIRC and GSE29609. Subsequently, this study investigated the association between disulfidptosis-related NMF signatures and the prognosis of patients with KIRC using Kaplan-Meier (K-M) analysis and univariate Cox regression. Cutoff values for different NMF clusters were determined utilizing the “survminer” R package, aiming to elucidate the link between disulfidptosis-related gene signatures and clinical outcomes in patients with KIRC.

### Immunotherapeutic analyses for NMF disulfidptosis-related subtypes

The TIDE database offers an integrated analysis of immune dysfunction and exclusion mechanisms in tumor immune evasion, aiding in predicting immunotherapy responses (35). The TIDE database was utilized, and logistic regression analysis was performed to assess immunotherapy response among different disulfidptosis-mediated TME patient subgroups in the TCGA-KIRC and GSE29609 datasets. This study then evaluated the likelihood of implementing immunotherapy responses for specific disulfidptosis-related NMF subtypes.

### Multiplex immunohistochemistry assay

Multiple immunohistochemistry (mIHC) was performed to detect three different antibodies on tissue sections. The primary antibodies used were rabbit monoclonal (1:500 dilution, ab186754, EPR15827(B); Abcam) to DSTN, rabbit monoclonal (1:200 dilution, ab76289, EP2405Y; Abcam) to FLNA, and rabbit monoclonal antibody (1:200 dilution, ab133616, EPR6855; Abcam) to CD4. The cancerous or adjacent normal tissues used for mIHC experiments were obtained from archived paraffin-embedded surgical specimens of patients with KIRC who provided prior informed consent. Employing the TSA method, the primary antibodies were stained. The procedure involved deparaffinization, antigen repair, labeling, inactivation of endogenous peroxidases, and antigen blocking on the first day. The first primary antibody, CD4, was applied, incubating overnight at 4°. On the second day, after treatment with goat antirabbit IgG (H+L; 1:50 dilution) labeled with horseradish peroxidase, Cyanine 5 Tyramide was used for detection. The steps were then repeated with DSTN or FLNA primary antibodies, incubating overnight at 4°. On the third day, following treatment with goat anti-rabbit IgG (H+L; 1:50 dilution) labeled with horseradish peroxidase, the sections were incubated with fluorescein tyramide working solution. Finally, nuclei were stained with DAPI.

### Cell culture, transfection, and cell scratch assay

The human KIRC cells (786-O) were cultured in RPMI-1640 (BI, Israel) supplemented with 1% streptomycin and penicillin, along with 10% fetal bovine serum (BI, Israel). siRNA transfection utilized riboFECT™ CP (RiboBio, China). The target sequences for TNFRSF1A-siRNA were as follows: GAACCTACTTGTACAATGA. Cultured cells were grown to 95% confluence in 24-well dishes. Subsequently, scratches were vertically created in each well using a 10-μL lance tip. Cells were then washed thrice with phosphate-buffered saline to remove any shed cells. To assess the trauma area at the scratch areas, photographs of the si-TNFRSF1A group and the normal group were captured at 0 and 24 hours, followed by the analysis of the resulting images using ImageJ software.

### Statistical analysis

R version 4.2.1 was employed for statistical analysis. To evaluate differences in continuous and categorical variables within cellular subgroups, various tests (Mann-Whitney U test, t-test, Kruskal-Wallis test, and log-rank test) were conducted. Furthermore, to compare distinct characteristics of disulfidptosis-mediated TME subgroups in KIRC, relevant disulfidptosis-related regulators and TME-related genes were obtained from prior literature. The “pheatmap” R package was utilized to visualize the NMF cluster. Statistical significance was determined at a threshold p-value of less than 0.05, indicating significant differences.

## Results

### Landscape of disulfidptosis regulators in the TME of KIRC

**Figure 1:**
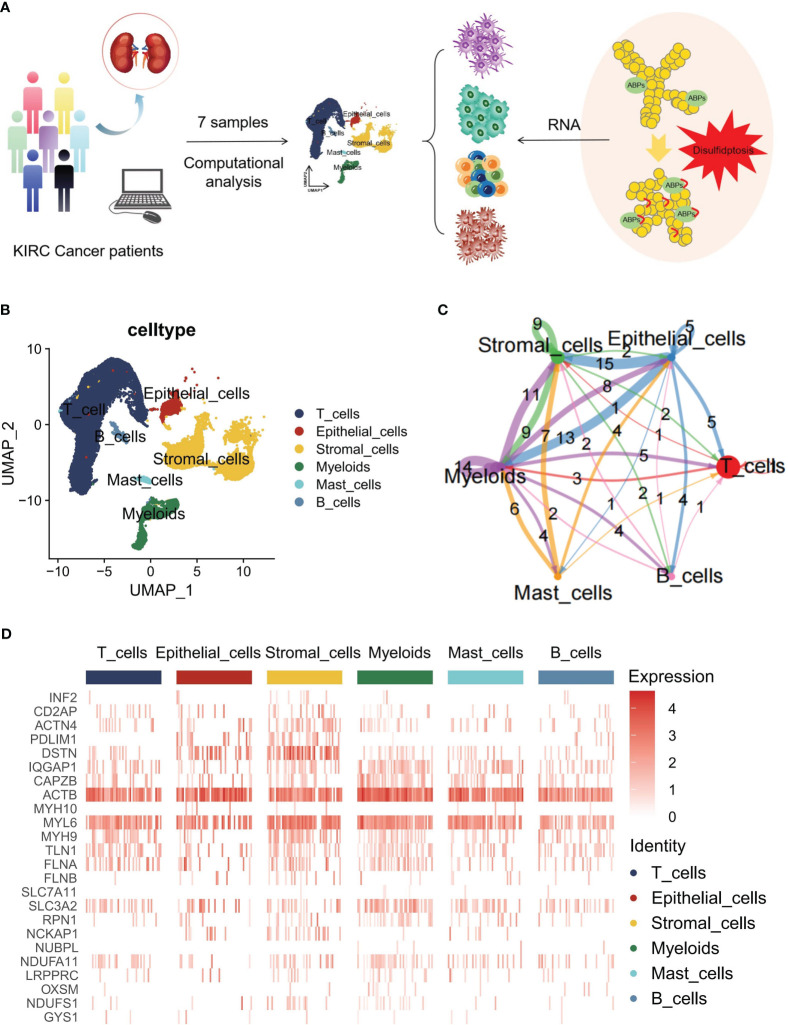
Overview of disulfidptosis regulators in the single-cell data for kidney renal clear cell carcinoma (KIRC). **(A)** Flowchart of the workflow used in this study. **(B)** Main cell type annotations using the Seurat uniform manifold approximation and projection (UMAP) plot of 41,784 cells. **(C)** Cell-cell communications between the main cell types by CellChat analysis. **(D)** Heatmap distribution of disulfidptosis regulators in T cells, epithelial cells, stromal cells, myeloids, mast cells, and B cells.

A concise flowchart was presented to illustrate the exploration of disulfidptosis regulators based on scRNA-seq data from patients with KIRC (**Figure 1A**). The GSE210038 dataset encompassed 41,784 TME cells from 7 patients with KIRC. Following a single-cell analysis workflow, these cells were annotated into major cell types—epithelial cells, stromal cells, myeloids, mast cells, B cells, and T cells (**Figure 1B**). Additionally, CellChat analysis revealed intercellular interactions among different cell types (**Figure 1C**). Furthermore, a heatmap was generated to visualize the expression differences of 24 disulfidptosis regulators across diverse cell types (**Figure 1D**).

### Novel disulfidptosis-mediated fibroblasts contributed to the TME of KIRC

**Figure 2:**
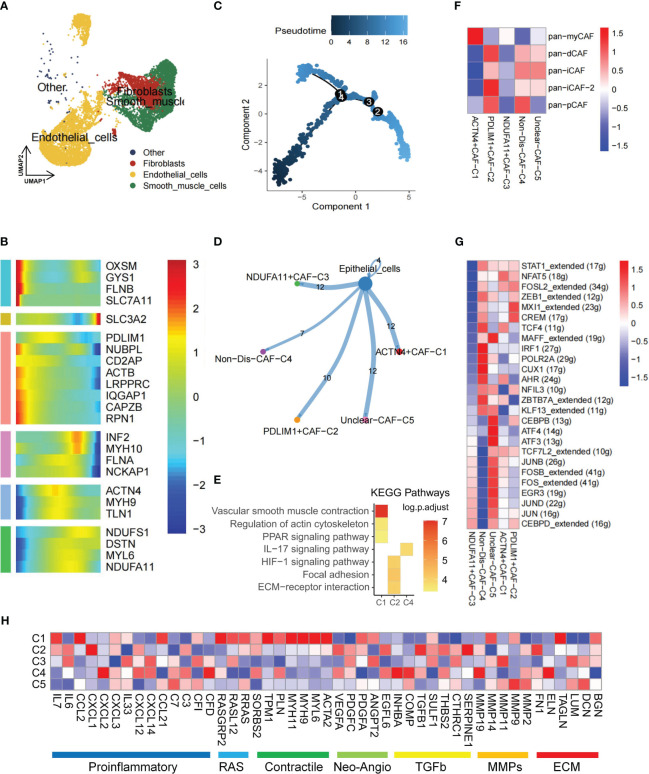
Disulfidptosis regulators modifying the features of fibroblast cells. **(A)** Presence of fibroblasts in stromal cells. **(B)** Heatmap of pseudotime trajectory analysis revealing the role of disulfidptosis-related genes in fibroblast cells (2,398 cells). **(C)** Trajectory analysis of fibroblast cells. **(D)** Cell-cell communications from disulfidptosis-related fibroblast subgroups to epithelial cells. **(E)** Activation of KEGG signaling pathways by the main disulfidptosis fibroblast subgroups, as depicted in the heatmap based on differentially expressed genes (DEGs; p < 0.05). **(F)** Correlation between different disulfidptosis-related fibroblast subgroups and different cancer-associated fibroblasts (CAFs) cluster characteristics (p < 0.05). **(G)** Comparison of activities of transcription factors (TFs) among the five disulfidptosis-related fibroblast subgroups, illustrated in the heatmap based on the average area under the curve (AUC) values (Kruskal-Wallis test, p < 0.001). The activity of TFs was evaluated using AUCell. **(H)** Heatmap showing the different average expression levels of common signaling pathway genes in the 5 disulfidptosis-related fibroblast subgroups, including Proinflammatory, RAS, Contractile, Neo-Angio, TGFb, MMPs, and ECM.

The TME is known to be a complex network where stromal cells play a crucial role by releasing various growth factors, cytokines, and signaling molecules. These molecules, such as platelet-derived growth factor, fibroblast growth factor, transforming growth factor-β (TGF-β), and interleukin (IL)-6, bind to receptors on tumor cells, promoting tumor growth, differentiation, and metastasis (36). Stromal cells encompass various types, including fibroblasts, endothelial cells, smooth muscle cells, and others (**Figure 2A**). CAFs in the KIRC TME are often linked with poor prognosis and resistance to immune checkpoint inhibitor therapy (37). To gain insights into the development and dynamics of the TME, pseudotime analysis was employed, which has proven to be a valuable tool in studying cell development, stem cell differentiation, and TME processes (38). By utilizing pseudotime analysis, the essential role of disulfidptosis regulators in the developmental trajectory of TME fibroblasts was unraveled. Specifically, genes related to disulfidptosis—OXSM, GYS1, FLNB, SLC7A11—were expressed at the beginning of the developmental process, while SLC3A2 expression was observed toward the end (**Figures 2B, C**). These genes may act as crucial mediators in TME subpopulation development and differentiation. Furthermore, intercellular communication and ligand-receptor interactions between subpopulations and tumor cells significantly influence tumor progression (39). CellChat analysis revealed varying ligand-receptor pairs links among clusters—ACTN4+CAF-C1 (n=466), PDLIM1+CAF-C2 (n=389), NDUFA11+CAF-C3 (n=266), Non-Dis-CAF-C4 (n=320), and Unclear-CAF-C5 (n=501)—and epithelial cells. Notably, disulfidptosis-mediated CAFs clusters exhibited stronger communication with tumor cells (**Figure 2D**).

Additionally, based on DEGs, KEGG enrichment analysis indicated associations of the ACTN4+CAF-C1 cluster with vascular smooth muscle contraction, regulation of actin cytoskeleton, and the PPAR signaling pathway. Meanwhile, the PDLIM1+CAF-C2 cluster showed involvement in the HIF-1 signaling pathway, focal adhesion, and ECM-receptor interaction (**Figure 2E**, **Supplementary Table S2**). Previous research underscores the role of iCAF in tumor progression and its influence on immune infiltration in bladder and breast cancers (40). Using Pan-CAF signatures from prior literature (41), a strong correlation existed between PDLIM1+CAF-C2 cell cluster scores and the pan-inflammatory CAF-2 (pan-iCAF-2; **Figure 2F**).

TFs play a pivotal role in either promoting or repressing downstream genes by recognizing specific DNA sequences. In renal cancer, TFs activation closely relates to somatic gene mutation and tissue-specific cancer risk (42). SCENIC analysis revealed distinct expression patterns of 26 TFs among the five clusters. Notably, TFs such as JUNB, FOS, EGR3, JUND, JUN, and CEBPD were upregulated in the NDUFA11+CAF-C3 cluster (**Figure 2G**). Given that JunB had a role in promoting cell invasion and angiogenesis in VHL-deficient RCC (43), we hypothesize that a close association between this CAFs cluster and tumor invasion. Finally, pathway heatmaps highlighted significant differences in gene expression levels, particularly in the contractile and RAS pathways, among ACTN4+CAF-C1, NDUFA11+CAF-C3, and unclear-CAF-C5 clusters (**Figure 2H**).

### Disulfidptosis-mediated macrophages exhibit distinct metabolism features

**Figure 3:**
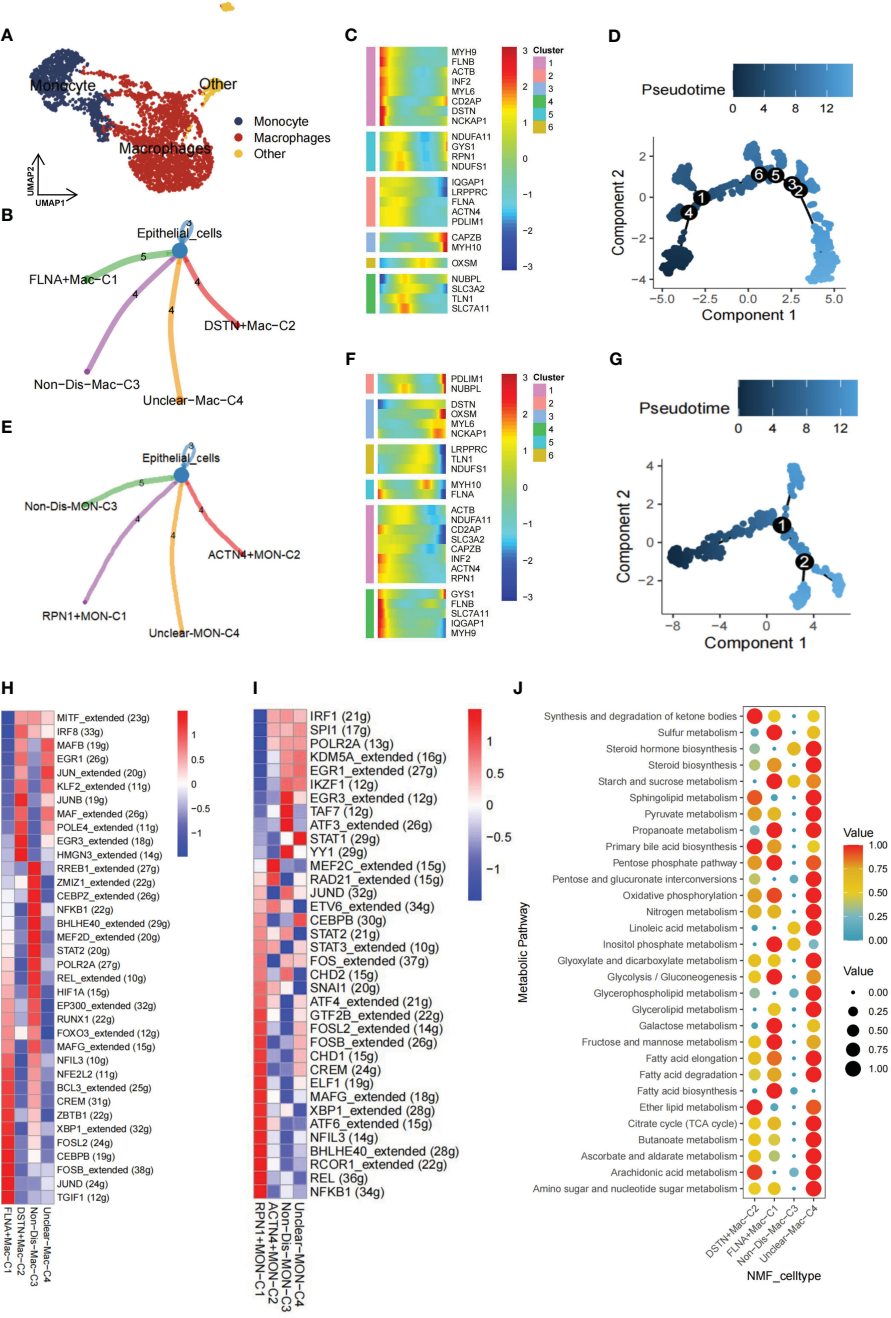
Nonnegative matrix factorization (NMF) clusters of disulfidptosis regulators for tumor-associated myeloid cells. **(A)** Myeloid cells are further subpopulated, and they include monocytes and macrophages. **(B)** Cell-cell communications from disulfidptosis-related macrophages to epithelial cells. **(C)** Heatmap of pseudotime trajectory analysis revealing the role of disulfidptosis-related genes in macrophages (1,841 cells). **(D)** Trajectory analysis for macrophages. **(E)** Cell-cell communications from disulfidptosis-related monocytes to epithelial cells. **(F)** Heatmap of pseudotime trajectory analysis revealing the role of disulfidptosis-related genes in monocytes (682 cells). **(G)** Trajectory analysis for monocytes. Comparison of TF activities among the four disulfidptosis-related macrophages subgroups **(H)** and four disulfidptosis-related monocyte subgroups **(I)**, illustrated in the heatmap based on the average area under the curve (AUC) values (Kruskal-Wallis test, p < 0.001). TF activity was evaluated using AUCell. **(J)** Heatmap showing significantly different activity of 30 metabolic signaling pathways scores by scMetabolism analysis for 1,841 cells in four disulfidptosis-related macrophage subgroups (Kruskal-Wallis test, p < 0.001).

Within the TME, myeloid cells play a crucial role in both innate and adaptive immunity and demonstrate significant heterogeneity (44). To delve into the functionality of disulfidptosis regulators within myeloid cells, this population was meticulously classified into distinct subgroups—comprising macrophages, monocytes, and other cellular entities (**Figure 3A**). Notably, our NMF clustering of macrophages uncovered four major clusters: FLNA+Mac-C1 (n=556), DSTN+Mac-C2 (n=326), Non-Dis-Mac-C3 (n=258), and Unclear-Mac-C4 (n=697). CellChat analysis revealed diverse ligand-receptor interactions between these clusters and epithelial cells (**Figure 3B**). Pseudotime analysis underscored the crucial involvement of disulfidptosis regulators in shaping the trajectory of TME macrophages (**Figures 3C, D**).

Similarly, for monocytes, CellChat analysis delineated distinct ligand-receptor connections between clusters: RPN1+MON-C1 (n=127), ACTN4+MON-C2 (n=100), Non-Dis-MON-C3 (n=220), and Unclear-MON-C4 (n=235) in relation to epithelial cells (**Figure 3E**). Monocyte pseudotime analysis mirrored the trends observed in macrophages (**Figures 3F, G**). Furthermore, SCENIC analysis of macrophages and monocytes unveiled unique activation patterns of potential TFs within the FLNA+Mac-C1, DSTN+Mac-C2, RPN1+MON-C1, and ACTN4+MON-C2 clusters (**Figures 3H, I**).

As the TME is closely associated with tumor metabolism and because tumors establish acidic and hypoxic environments by exploiting resources from the surrounding microenvironment, tumor cells undergo metabolic reprogramming to fulfill the specific demands necessary for their growth, proliferation, and survival (45). Lastly, to assess the relationship between the disulfidptosis-mac cluster with metabolic pathways. GSVA and scMetabolism were utilized to gauge single-cell metabolic activity, revealing notable differences in 30 metabolic pathways among the four clusters (**Figure 3J**). Particularly, the FLNA+Mac-C1 cluster exhibited heightened activation of sulfur metabolism, suggesting its potential significance in regulating disulfidptosis.

### Novel disulfidptosis-mediated B/T cell immune response in KIRC

**Figure 4:**
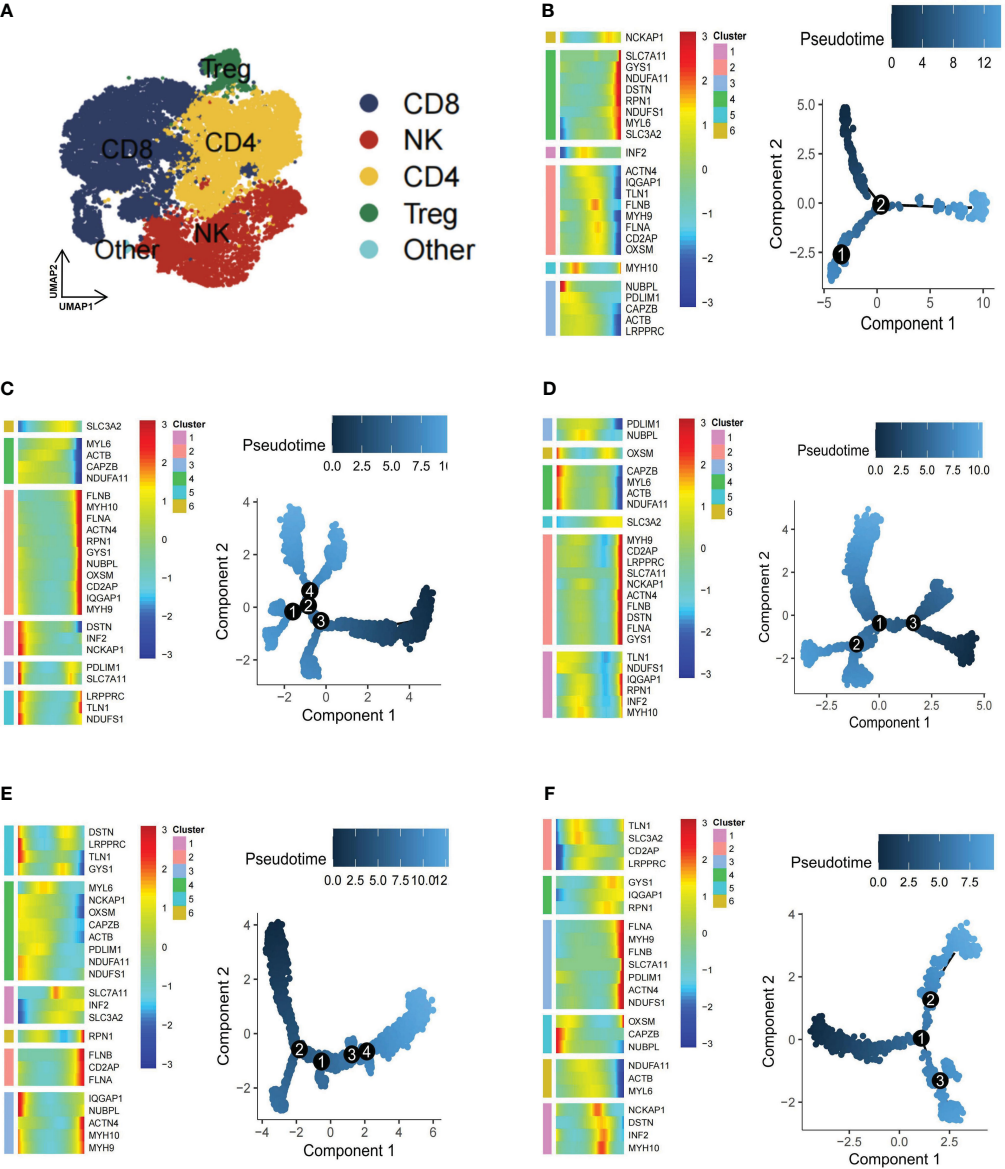
Pseudotime trajectory analysis of disulfidptosis-related B cells and T cells subgroups of kidney renal clear cell carcinoma (KIRC). **(A)** Four main types of T cells (CD4^+^ T, CD8^+^ T, natural killer [NK], and Treg cells). Heatmap of pseudotime trajectory analysis of disulfidptosis-related B-cell and T-cell subgroups of KIRC, including B cells **(B)**, CD4^+^ T cells **(C)**, CD8^+^ T cells **(D)**, NK cells **(E)**, and Treg cells **(F)**.

Immune cells are fundamental in protecting the body against pathogens and influencing various disorders, including inflammation, hematological conditions, and tumors (46). The TME, an intricate ecosystem, hosts diverse immune cell types, each intricately involved in modulating tumors (47). To gain a comprehensive understanding of the dynamics of immune cells in TME, from a total of 25,390 T cells, subpopulations—CD4^+^ T cells (n=7818), CD8^+^ T cells (n=10796), natural killer (NK) cells (n=5621), and Treg cells (n=1104)—were further classified (**Figure 4A**). Subsequently, CD4^+^ T cells exhibited five subgroups: DSTN+CD4T-C1 (n=1624), FLNA+CD4T-C2 (n=509), Other-Dis-CD4T-C3 (n=4909), Non-Dis-CD4T-C4 (n=159), and Unclear-CD4T-C5 (n=531). The CD8^+^ T cells were further divided into NDUFA11+CD8T-C1 (n=3208), FLNA+CD8T-C2 (n=1616), Other-Dis-CD8T-C3 (n=5009), Non-Dis-CD8T-C4 (n=502), and Unclear-CD8T-C5 (n=358). Similarly, NK and Treg cells were also identified to exist as 5 subgroups, including DSTN+NKT-C1 (n=531), TLN1+NKT-C2 (n=260), IQGAP1+NKT-C3 (n=188), Other-Dis-NKT-C4 (n=4447), Non-Dis-NKT-C5 (n=178), NDUFA11+Treg-C1 (n=375), FLNA+Treg-C2 (n=157), TLN1+Treg-C3 (n=125), Other-Dis-Treg-C4 (n=355), and Non-Dis-Treg-C5 (n=85). Among 897 B cells, 6 subgroups were identified to exist: NDUFA11+B-C1 (n=196), FLNA+B-C2 (n=174), CAPZB+B-C3 (n=162), TLN1+B-C4 (n=139), MYH9+B-C5 (n=110), and Unclear-B-C6 (n=113). Pseudotime analysis highlighted the critical role of disulfidptosis regulators in the trajectory process of TME B cells, CD4^+^ T cells, CD8^+^ T cells, NK cells, and Treg cells (**Figures 4B–F**).

**Figure 5:**
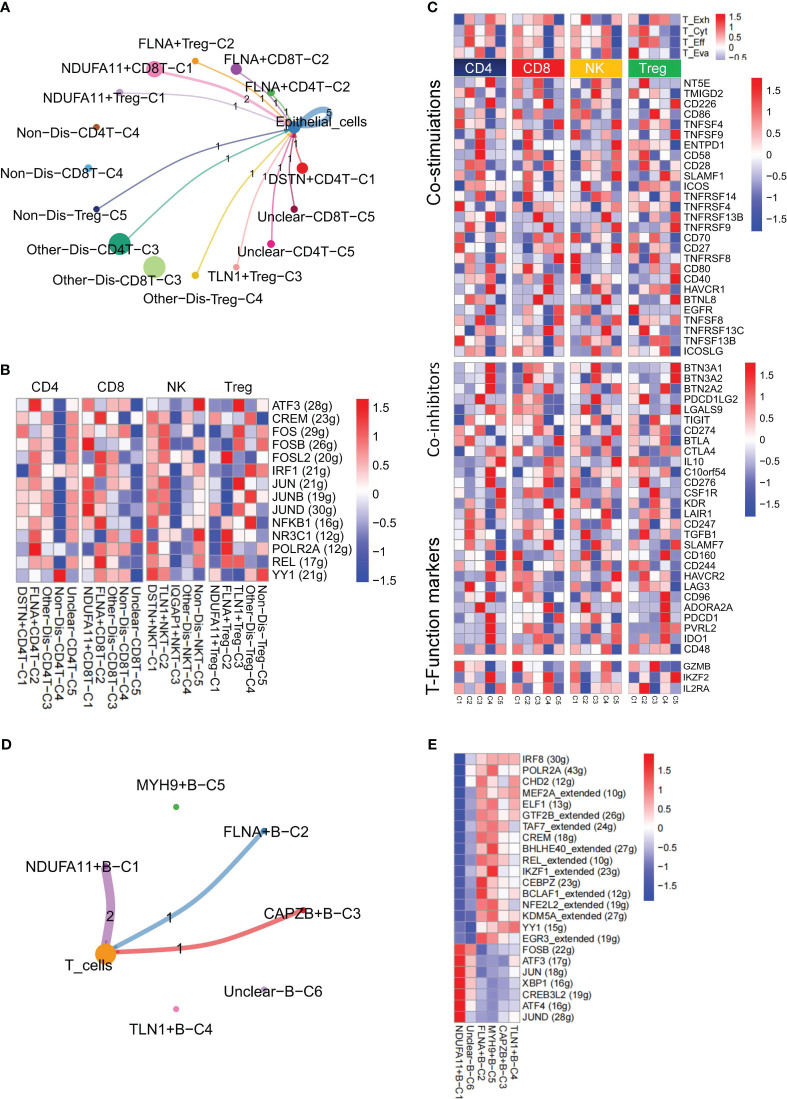
Nonnegative matrix factorization (NMF) clusters of disulfidptosis regulators for B cells and T cells. **(A)** Cell-Cell communications from disulfidptosis-related T cells subgroups to epithelial cells. **(B)** Comparison of TFs activities among the disulfidptosis-related subgroups in CD4^+^ T cells, CD8^+^ T cells, NK cells, and Treg cells, illustrated in the heatmap based on the average area under the curve (AUC) values (Kruskal-Wallis test, p < 0.001). TFs activity was evaluated using AUCell. **(C)** Heatmap showing significantly different features among disulfidptosis-related T cells subgroups of CD4^+^ T, CD8^+^ T, NK cells, and Treg cells, including T exhaustion score, T cytotoxic score, T effector score, and T evasion score, as well as some immune co-stimulators, co-inhibitors, and T-Function markers (Kruskal-Wallis test, p < 0.001). **(D)** Cell-Cell communications from disulfidptosis-related B cells subgroups to T cells. **(E)** Comparison of TFs activities among disulfidptosis-related B cells subgroups, illustrated in the heatmap based on the average AUC values (Kruskal-Wallis test, p < 0.001). TFs activity was evaluated using AUCell.

Utilizing CellChat, this study unveiled a diverse range of ligand-receptor interactions between disulfidptosis-related T cell clusters and tumor epithelial cells (**Figure 5A**). SCENIC analysis revealed substantial differences in the expression of TFs among CD4^+^ T cells, CD8^+^ T cells, NK cells, and Treg cells within these disulfidptosis clusters (**Figure 5B**). Additionally, to evaluate the collective effect of disulfidptosis-related T cell subgroups on T cells, notable differences were observed in the average expression of immune genes associated with co-stimulation, co-inhibition, and functional markers. Variations were also noted in the T exhaustion score, T cytotoxic score, T effector score, and T evasion score among these four disulfidptosis-related T cell subgroups (**Figure 5C**). Furthermore, distinctive ligand-receptor interactions were observed between disulfidptosis-related B cells and T cells (**Figure 5D**). The heatmap illustrated distinct TFs among the disulfidptosis-related B cell clusters (**Figure 5E**). These findings strongly indicate robust heterogeneity among disulfidptosis-related T/B cells. Given the pivotal role of CD4^+^ T cells in tumor immune responses (46), our focus was directed toward investigating the significance of disulfidptosis-mediated CD4^+^ T cells.

### Disulfidptosis-mediated TME patterns contributed to the KIRC prognosis and immunotherapy

**Figure 6:**
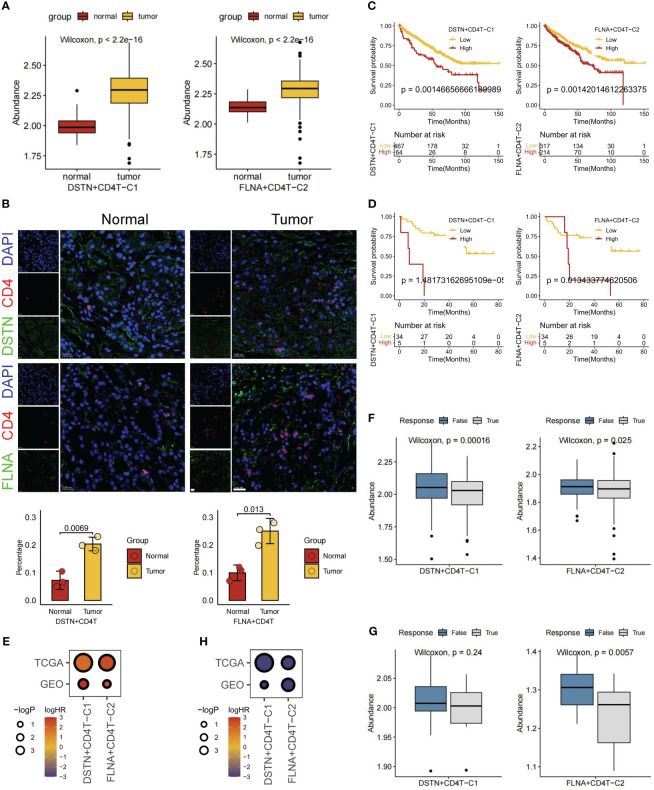
Overall expression, prognosis, and immunotherapy response of disulfidptosis-related cell types. **(A)** Based on The Cancer Genome Atlas (TCGA) database for normal and tumor samples to compare the gene set variation analysis (GSVA) scores of two disulfidptosis-related CD4^+^ T cell subgroups. **(B)** Multiple immunohistochemistry shows the localization of DSTN^+^ CD4^+^ T cells and FLNA^+^ CD4^+^ T cells in tumor or normal tissues of patients with KIRC (scale bar, 20 μm). Survival analysis of two disulfidptosis-related CD4^+^ T cell subgroups based on TCGA **(C)** and Gene Expression Ominbus (GEO) **(D)** databases. **(E)** Bubble heatmap of univariate Cox regression analysis (survival). Immunotherapy response analysis of two disulfidptosis-related CD4^+^ T cell subgroups based on TCGA **(F)** and GEO **(G)** databases. H Bubble heatmap of logistic regression analysis (immunotherapy response).

For a comprehensive evaluation of disulfidptosis in KIRC at the bulk level, FindAllMarkers was employed to compute all DEGs mediated by disulfidptosis in TME cells (**Supplementary Table S3**). Subsequently, using GSVA, the scores of different cell subgroups in tumor (n = 531) and normal (n = 76) samples were calculated from the TCGA-KIRC cohort. Consequently, significant score differences between tumor and normal samples were observed within two disulfidptosis-related CD4^+^ T cell subgroups, namely, DSTN+CD4T-C1 and FLNA+CD4T-C2 (**Figure 6A**). To validate the differential expression and distribution of these two CD4^+^ T cell subgroups within the tumor and adjacent nontumor regions, this study conducted multiplexed immunohistochemistry (mIHC) assays. Statistical analysis further indicated a significantly higher proportion of these CD4^+^ T cell subpopulations in the tumor samples than in the adjacent nontumor samples (p < 0.05; **Figure 6B**). Subsequent survival analysis, utilizing information from 531 patients with KIRC in the TCGA-KIRC and GSE29609 public cohorts, revealed that patients with low expression of DSTN+CD4T-C1 and FLNA+CD4T-C2 subgroups exhibited better prognoses (**Figures 6C, D**). Univariate Cox regression analysis consistently showed prognostic significance for patients in both subgroups (**Figure 6E**). Furthermore, the TIDE database was utilized to investigate the immune response of these two disulfidptosis-related CD4^+^ T cell subgroups in patients undergoing immunotherapy. TIDE scores indicated that patients with low expression in these subgroups exhibited better responses to immunotherapy (**Figures 6F, G**). Logistic regression analysis also demonstrated consistent immunotherapy response among patients in both subgroups (**Figure 6H**). Our ongoing investigation aims to explore the interaction between these two disulfidptosis-related CD4^+^ T cell subgroups and tumor cells, along with their mechanisms of exerting pro-oncogenic functions.

### Disulfidptosis-mediated TME of KIRC enhances intercellular communication

**Figure 7:**
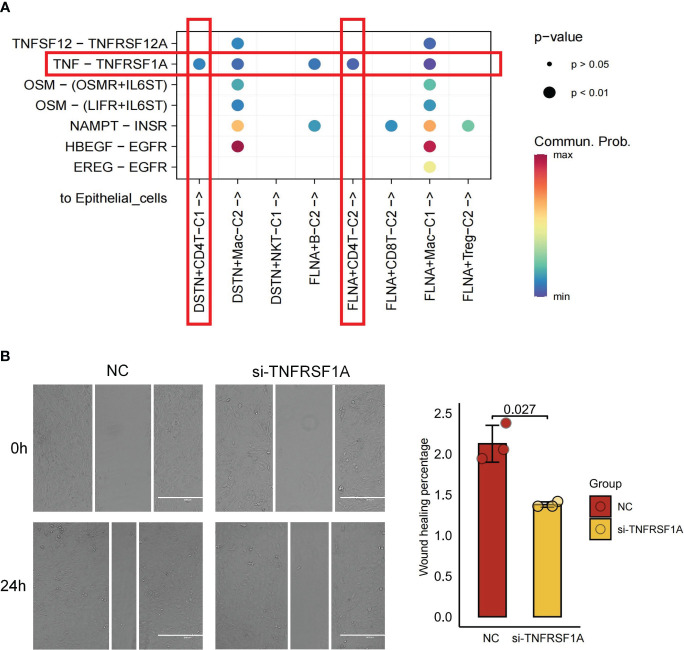
Cell-cell communications from disulfidptosis-related tumor microenvironment (TME) subgroups to epithelial cells. **(A)** Receptor-ligand pairs from DSTN/FLNA-related TME subgroups to epithelial cells. **(B)** Scratch healing assay suggesting a significant reduction in 796-O cell migration in silencing TNFRSF1A (scale bar, 400 μm; p = 0.027). All data are expressed as mean ± standard deviation of three independent experiments.

The interaction between tumor cells and cells in the TME significantly contributes to tumor progression (48). Through cellchat analysis, we aimed to elucidate the comprehensive ligand-receptor interactions between disulfidptosis-mediated TME subgroups and tumor epithelial cells, involving various signaling pairs such as TNFSF12-TNFRSF12A, TNF-TNFRSF1A, OSM-(OSMR+IL6ST), OSM-(LIFR+IL6ST), HBEGF-EGFR, and EREG-EGFR. Of particular interest, the interactions of signaling pairs TNFSF12-TNFRSF12A mediated the interaction of both DSTN+CD4T-C1 and FLNA+CD4T-C2 subgroups with tumor epithelial cells (**Figure 7A**). To further investigate this interaction’s functional significance, knockdown experiments targeting TNFRSF12A were conducted using siRNA in the KIRC cell line 786-O. Remarkably, after TNFRSF12A knockdown, significant inhibition of tumor cell migration was observed (p < 0.05; **Figure 7B**). These findings suggest that interactions between the DSTN+CD4T-C1 and FLNA+CD4T-C2 subgroups with tumor cells through the TNFSF12-TNFRSF12A signaling pairs play a crucial role in promoting tumor migration. This suggests that the progression of KIRC could be influenced by the interplay between disulfidptosis-mediated TME and tumor cells.

## Discussion

Disulfidptosis, a novel manifestation of disulfide stress-mediated PCD, has garnered attention due to its correlation with cancer pathogenesis (26, 49). Notably, studies using disulfidptosis scores have identified the CD96 gene as an independent prognostic marker for esophageal squamous cell carcinoma (ESCC). Knocking down CD96 not only significantly suppresses ESCC cell proliferation but also promotes apoptosis (50). Prognostic markers consisting of disulfidptosis-related lncRNAs can predict the survival of patients with different colorectal cancers and the use of targeted therapies and immunotherapies accordingly to the patient’s condition (51). Additionally, a prognostic model (Enet), based on disulfidptosis-related genes, aids in predicting the prognosis for patients with thyroid carcinoma (52). Yet, limited research exists on the role of disulfidptosis in cancer at the single-cell level. This study performed a comprehensive investigation into the regulatory factors for disulfidptosis in various cell types within the KIRC TME, exploring different disulfidptosis-mediated TME subgroups’ functions and determining cell-cell interactions between these subtypes. This study aimed to provide detailed insights into how these distinct TME subgroups influenced by disulfidptosis influence the prognosis of patients with KIRC.

Tumor tissue consists of tumor cells and various stromal cells, together forming a complex TME that plays a crucial role in tumor growth, invasion, and immune evasion (53). Components of tumor stroma induce inflammatory responses and angiogenesis, promoting tumorigenesis (54). Although immune cells, including cytotoxic T cells, traditionally exhibit antitumor effects (55), distinct immune cell subpopulations may harbor both protumor and antitumor characteristics, possibly influenced by altered metabolic programs within the TME (56). The interaction between tumor cells and the TME can positively or negatively regulate tumor growth (57, 58). This study unraveled various disulfidptosis regulatory patterns in the TME of KIRC, involving fibroblasts, B cells, myeloid cells, and T cells, suggesting extensive communication with tumor epithelial cells. Moreover, CellChat analysis unveiled ligand-receptor pairs mediating communication between tumor epithelial cells and disulfidptosis-related TME subgroups, such as TNFSF12-TNFRSF12A, TNF-TNFRSF1A, OSM-(OSMR+IL6ST), OSM-(LIFR+IL6ST), HBEGF-EGFR, and EREG-EGFR.

Compared to normal fibroblasts, CAFs increase the production of extracellular matrix proteins (59), promoting the secretion of factors that support tumor growth. Additionally, CAFs impede antitumor therapy efficacy by remodeling the extracellular matrix, establishing a barrier hindering drug or immune cell infiltration into tumor tissues (60). CAFs activation during cancer progression involves pathways including recruitment and activation of resident fibroblasts, epithelial-mesenchymal transition (EMT), endothelial-mesenchymal transformation (EndMT), and differentiation of bone marrow-derived mesenchymal cells (61–65). Through secretion of chemokines and effector molecules such as c-x-c chemokine ligand 5 (CXCL5), IL-1β, matrix metalloproteinases (MMPs), and collagen, CAFs contribute to immunosuppression and tumor angiogenesis (66). Moreover, different CAFs subpopulations modulate gene expression, regulate regulatory T-cell accumulation, and influence immune and cancer-related pathways by interacting with tumor cells (67). Despite five identified CAFs subtypes, pan-myCAF, pan-dCAF, pan-iCAF, pan-iCAF-2, and pan-pCAF (41). Limited research exists on the potential regulatory role of disulfidptosis mechanisms in CAFs. Our study revealed that disulfidptosis-related fibroblasts exhibit extensive communication with tumor epithelial cells compared to non-disulfidptosis-mediated fibroblasts.

This study revealed a strong correlation between the expression of ACTN4+CAF-C1 and increased levels of MMPs, particularly MMP9 and MMP14. This suggests that CAFs potentially contribute to the formation of the TME conducive to tumor metastasis through the secretion of MMP9 and MMP14 (68, 69). Furthermore, KEGG pathway analysis highlighted the involvement of CAFs in various pathways such as vascular smooth muscle contraction, actin cytoskeleton regulation, and the PPAR signaling pathway. Additionally, PDLIM1+CAF-C2 displayed heightened expression levels of SERPINE1, CTHRC1, THBS2, SULF1, TGFB1, FN1, and BGN. Pathway analysis also indicated the participation of CAFs in the HIF-1 signaling pathway, focal adhesion, and ECM-receptor interaction. CAFs may participate in the HIF-1 signaling pathway and ECM pathways through the secretion of TGFB1, FN1, and BGN (70–72). Tumors are known to exploit ECM remodeling to create microenvironments conducive to tumorigenesis and metastasis (73). Consequently, we suggests that disulfidptosis-mediated CAFs may potentially enhance tumor invasion and metastasis by influencing tumor cell motility and ligand-receptor interactions.

CAFs release pro-inflammatory cytokine IL-1 and the chemokine CXCL1 to recruit macrophages, driving their differentiation into pro-tumorigenic macrophages (M2-like TAMs) (74). Macrophages have a crucial role in immune regulation and controlling inflammation. However, in the context of cancer, their functionality becomes altered. Upon interaction with the TME, macrophages induce immunosuppression, which hampers the effector T cell response (75). Metabolic processes profoundly influence TAMs, regulating cancer development and immune responses involving glucose, glutamine, and fatty acid metabolism (76). Under glucose-deficient conditions, elevated expression of SLC7A11 leads to rapid depletion of intracellular NADPH, causing abnormal accumulation of disulfides such as cysteine, which triggers disulfide stress and rapid cell death. Through NMF clustering, extensive crosstalk occurred between disulfidoptosis-mediated macrophages and tumor cells. Notably, disulfidoptosis-mediated macrophages, particularly the FLNA+Mac-C1 subtype, exhibit significant activation of metabolic pathways including sulfur metabolism, the pentose phosphate pathway, oxidative phosphorylation, glycolysis, and gluconeogenesis. Furthermore, the study identified interactions between disulfidoptosis-mediated CD4^+^ T cells, CD8^+^ T cells, Treg cells, B cells, and tumor cells were identified, each demonstrating distinct functional characteristics. These findings collectively underscore the significant roles of disulfidoptosis-related macrophages and T cells in tumor biology.

Subsequently, this study investigated the TF activity within each disulfidoptosis-related subpopulation. Understanding TFs is crucial for deciphering gene regulatory networks, cellular development, and tumor occurrences (77). Single-cell level analysis in KIRC revealed distinct TF characteristics among fibroblasts, macrophages, monocytes, B cells, and T cell subtypes. NDUFA11+CAF-C3 among CAFs exhibited activation of several TF genes including CEBPD, JUN, JUND, EGR3, FOS, FOSB, and JUNB. Previous studies have highlighted the downregulation of CEBPD in RCC (78) and the association of FOS and JUNB with renal cancer (43, 79). Similarly, among monocytes, the RPN1+MON-C1 subtype displayed the activation of multiple TFs such as NFKB1, REL, RCOR1, BHLHE40, NFIL3, ATF6, XBP1, MAFG, ELF1, CREM, CHD1, FOSB, FOSL2, and GTF2B. Hong et al. observed the activation of the transcriptional repressor BHLHE40 in KIRC. This activation inhibits the mTOR inhibitor DEPTOR, contributing to tumor growth and drug resistance (80). Additionally, ATF6 amplifies apoptosis in sunitinib-resistant KIRC cells through the endoplasmic reticulum stress pathway, thereby influencing tumor progression (81).

In macrophages, higher activity of TFs including TGIF1, JUND, FOSB, CEBPB, FOSL2, XBP1, ZBTB1, CREM, BCL3, NFE2L2, NFIL3, EP300, RUNX1, and HIF-1A was observed in the FLNA-mac-C1 subtype. BCL3 and EP300 as prognostic factors for KIRC (82, 83), and upregulated RUNX1 is closely associated with renal cancer progression (84). Moreover, macrophage HIF-1α has been identified as an independent prognostic indicator for renal cancer, being associated with highly invasive or deteriorating renal tumors (85). This led to the hypothesis that a close association between the FLNA-mac-C1 subtype and cancer progression. Furthermore, diverse TF characteristics were noted for disulfidptosis-mediated B and T cell subgroups. In summary, disulfidptosis-mediated TME subgroups likely regulate distinct TF networks, reshaping the TME. Lastly, cellular network analysis revealed significant connectivity and communication between these disulfidptosis-mediated TME subgroups and tumor cells.

The effectiveness of tumor therapy heavily relies on the microenvironment, particularly the tumor immune microenvironment, intricately linked to treatment prognosis (86). Immunotherapy has recently emerged as a promising strategy for cancer treatment, becoming a pivotal component in many cancer treatment regimens (87). Its therapeutic mechanism involves reactivating the immunosuppressive environment caused by cancer cells and boosting the immune cell-mediated anti-tumor response (88). Chen et al. studied the tumor immune microenvironment infiltration characteristics of disulfidptosis-related genes in breast cancer and reported that TNFRSF14, among disulfidptosis-related genes, serves as a key regulatory gene. Targeting TNFRSF14 alongside immune checkpoint inhibition was observed to inhibit tumor proliferation and induce disulfidptosis in tumor cells (89). Alterations in characteristic tumor biomarkers are closely related to tumor occurrence and development (90). By detecting the expression levels of key biomarkers and taking into account the clinical characteristics of patients, it is important to predict the prognosis of patients (91). Furthermore, emerging nano-formulations and tumor antigen vaccines have demonstrated high specificity and efficiency (92, 93). Guan L et al. synthesized mesoporous organosilicon nanoparticles exhibiting robust antitumor abilities and biosafety (94). Nevertheless, because of the complexity of the TME, the development of more targeted drugs tailored to different patients remains necessary.

Understanding the complex intrinsic regulatory patterns of disulfidptosis in the TME of KIRC, a comprehensive analysis using bulk RNA-seq and scRNA-seq data from TCGA and GEO was conducted. This analysis aimed to investigate the relationship between different disulfidptosis-mediated TME subgroups, prognosis, and immunotherapy. Consequently, significant variations in patient prognosis were observed based on different disulfidptosis factors. Specifically, the integrated scores of DSTN+CD4T-C1 and FLNA+CD4T-C2 subgroups were upregulated in tumor tissues and associated with unfavorable prognosis. These findings were corroborated by employing mIHC on tissue sections from patients with KIRC to confirm the expression and location of these two cell subgroups. Numerous studies indicate that CD4^+^ T cells, a major T lymphocyte subpopulation, play a crucial role in tumor immunity and exhibit diverse functions (95). Notably, CD4^+^ T cells effectively impede tumor cell division by arresting their cell cycle at the G1/S phase, inhibiting tumor growth (96). Furthermore, an independent study noted that CD4^+^ T cells isolated from bladder tumors exhibited substantial cytotoxicity when cultured *in vitro*, inducing apoptosis in tumor cells (97). Our investigation revealed that that DSTN+CD4T and FLNA+CD4T cells interact with tumor cells through the TNF-TNFRSF1A signaling pair, thereby facilitating tumor migration. The TME might influence CD4^+^ T cells to produce cytokines with pro-tumorigenic functions, promoting tumor survival (98). Additionally, because of their plasticity, CD4^+^ T cells can convert into Treg cells secreting IL-10 and TGF-β, suppressing immune responses and aiding in tumor immune escape (99). These findings align with our conclusions and support the development of therapeutic strategies targeting these CD4^+^ T cell subsets associated with tumors.

However, it is important to note that this study was analyzed based on scRNA-seq data from public databases, and a self-test cohort of clinical samples is still needed to provide more realistic and accurate results. Besides, this study’s scope is confined to *in vitro* cellular experiments, susceptible to various confounding factors and biases. Further studies using flow cytometric analysis and sorting experiments and *in vivo* animal models are required to comprehensively investigate the interaction between these two cellular subpopulations and tumor cells through the TNF-TNFRSF1A signaling pair, as well as the potential influence of other cell-cell communications on tumor cells. The TNF-TNFRSF1A signaling pair strongly correlates with responses to and resistance against anti-PD-1 treatment (100). The results of the present study indicate significant differences in immune responses to immune checkpoint blockade therapy among patients from different subgroups, underscoring the crucial role of disulfidptosis in patients with KIRC and warranting further investigation. Therefore, this study emphasizes the importance of exploring disulfidptosis concerning prognosis and immunotherapy in patients with KIRC.

## Conclusion

For the first time, this study utilized the scRNA-seq analysis method to identify disulfidptosis-mediated TME subgroups. Additionally, combining bulk RNA-seq enabled us to elucidate the role of disulfidptosis-mediated cell-cell communication in regulating tumor growth and anti-tumor immune modulation.

## Data availability statement

The original contributions presented in the study are included in the article/**Supplementary Material**. Further inquiries can be directed to the corresponding authors.

## Ethics statement

The studies involving humans were approved by Ethics Committee of Binhai County People’s Hospital. The studies were conducted in accordance with the local legislation and institutional requirements. The participants provided their written informed consent to participate in this study.

## Author contributions

KX: Writing – original draft. DL: Writing – original draft. JQ: Data curation, Writing – review & editing. YZ: Data curation, Writing – review & editing. MZ: Data curation, Writing – review & editing. HZ: Investigation, Writing – review & editing. XH: Investigation, Writing – review & editing. JJ: Investigation, Writing – review & editing. ZZ: Writing – review & editing. HS: Writing – review & editing. GS: Writing – review & editing. HD: Supervision, Writing – review & editing. HL: Supervision, Writing – review & editing.

## References

[1] Siegel RL Miller KD Wagle NS Jemal A. Cancer statistics, 2023. CA Cancer J Clin (2023) 73:17–48. doi: 10.3322/caac.21763 36633525

[2] Wei JH Feng ZH Cao Y Zhao HW Chen ZH Liao B. Predictive value of single-nucleotide polymorphism signature for recurrence in localised renal cell carcinoma: a retrospective analysis and multicentre validation study. Lancet Oncol (2019) 20:591–600. doi: 10.1016/s1470-2045(18)30932-x 30880070

[3] Mao W Wang K Xu B Zhang H Sun S Hu Q. ciRS-7 is a prognostic biomarker and potential gene therapy target for renal cell carcinoma. Mol Cancer (2021) 20:142. doi: 10.1186/s12943-021-01443-2 34740354 PMC8570002

[4] Tang D Kang R Berghe TV Vandenabeele P Kroemer G. The molecular machinery of regulated cell death. Cell Res (2019) 29:347–64. doi: 10.1038/s41422-019-0164-5 PMC6796845 30948788

[5] Wang J Qi X Wang Q Wu G. The role and therapeutic significance of the anoikis pathway in renal clear cell carcinoma. Front Oncol (2022) 12:1009984. doi: 10.3389/fonc.2022.1009984 36249029 PMC9557223

[6] Zhou X Yao L Zhou X Cong R Luan J Wei X. Pyroptosis-related lncRNA prognostic model for renal cancer contributes to immunodiagnosis and immunotherapy. Front Oncol (2022) 12:837155. doi: 10.3389/fonc.2022.837155 35860590 PMC9291251

[7] Gu J He Z Huang Y Luan T Chen Z Wang J. Clinicopathological and prognostic value of necroptosis-associated lncRNA model in patients with kidney renal clear cell carcinoma. Dis Markers (2022) 2022:5204831. doi: 10.1155/2022/5204831 35664432 PMC9157284

[8] Cai Z He Y Yu Z Hu J Xiao Z Zu X. Cuproptosis-related modification patterns depict the tumor microenvironment, precision immunotherapy, and prognosis of kidney renal clear cell carcinoma. Front Immunol (2022) 13:933241. doi: 10.3389/fimmu.2022.933241 36211378 PMC9540508

[9] Liu X Nie L Zhang Y Yan Y Wang C Colic M. Actin cytoskeleton vulnerability to disulfide stress mediates disulfidptosis. Nat Cell Biol (2023) 25:404–14. doi: 10.1038/s41556-023-01091-2 PMC10027392 36747082

[10] Ren W Zhao W Cao L Huang J. Involvement of the actin machinery in programmed cell death. Front Cell Dev Biol (2020) 8:634849. doi: 10.3389/fcell.2020.634849 33634110 PMC7900405

[11] Zhang Y Chang SKC. Color and texture of surimi-like gels made of protein isolate extracted from catfish byproducts are improved by washing and adding soy whey. J Food Sci (2022) 87:3057–70. doi: 10.1111/1750-3841.16229 35708220

[12] Franklin-Tong VE Gourlay CW. A role for actin in regulating apoptosis/programmed cell death: evidence spanning yeast, plants and animals. Biochem J (2008) 413:389–404. doi: 10.1042/bj20080320 18613816

[13] Smertenko A Franklin-Tong VE. Organisation and regulation of the cytoskeleton in plant programmed cell death. Cell Death Differ (2011) 18:1263–70. doi: 10.1038/cdd.2011.39 PMC3172095 21566662

[14] Chen H Yang W Li Y Ma L Ji Z. Leveraging a disulfidptosis-based signature to improve the survival and drug sensitivity of bladder cancer patients. Front Immunol (2023) 14:1198878. doi: 10.3389/fimmu.2023.1198878 37325625 PMC10266281

[15] Yang L Liu J Li S Liu X Zheng F Xu S. Based on disulfidptosis, revealing the prognostic and immunological characteristics of renal cell carcinoma with tumor thrombus of vena cava and identifying potential therapeutic target AJAP1. J Cancer Res Clin Oncol (2023) 149:9787–804. doi: 10.1007/s00432-023-04877-x PMC11796798 37247081

[16] Xu WH Xu Y Wang J Wan FN Wang HK Cao DL. Prognostic value and immune infiltration of novel signatures in clear cell renal cell carcinoma microenvironment. Aging (Albany NY) (2019) 11:6999–7020. doi: 10.18632/aging.102233 31493764 PMC6756904

[17] Qi F Li J Qi Z Zhou B Yang B Zhang J. Modeling cross-talk of RNA modification enzymes reveals tumor microenvironment-associated clinical significance and immunotherapy prediction in hepatobiliary Malignancy. MedComm (2020) (2023) 4:e256. doi: 10.1002/mco2.256 37090117 PMC10113697

[18] Chevrier S Levine JH Zanotelli VRT Silina K Schulz D Bacac M. An immune atlas of clear cell renal cell carcinoma. Cell (2017) 169:736–749.e18. doi: 10.1016/j.cell.2017.04.016 28475899 PMC5422211

[19] Korman AJ Garrett-Thomson SC Lonberg N. The foundations of immune checkpoint blockade and the ipilimumab approval decennial. Nat Rev Drug Discovery (2022) 21:509–28. doi: 10.1038/s41573-021-00345-8 34937915

[20] Ma J Huang L Hu D Zeng S Han Y Shen H. The role of the tumor microbe microenvironment in the tumor immune microenvironment: bystander, activator, or inhibitor? J Exp Clin Cancer Res (2021) 40:327. doi: 10.1186/s13046-021-02128-w 34656142 PMC8520212

[21] Hu J Chen Z Bao L Zhou L Hou Y Liu L. Single-Cell Transcriptome Analysis Reveals Intratumoral Heterogeneity in ccRCC, which Results in Different Clinical Outcomes. Mol Ther (2020) 28:1658–72. doi: 10.1016/j.ymthe.2020.04.023 PMC7335756 32396851

[22] Hakimi AA Voss MH Kuo F Sanchez A Liu M Nixon BG. Transcriptomic profiling of the tumor microenvironment reveals distinct subgroups of clear cell renal cell cancer: Data from a randomized phase III trial. Cancer Discovery (2019) 9:510–25. doi: 10.1158/2159-8290.Cd-18-0957 PMC6697163 30622105

[23] Chen YP Yin JH Li WF Li HJ Chen DP Zhang CJ. Single-cell transcriptomics reveals regulators underlying immune cell diversity and immune subtypes associated with prognosis in nasopharyngeal carcinoma. Cell Res (2020) 30:1024–42. doi: 10.1038/s41422-020-0374-x PMC7784929 32686767

[24] Wu C Yang J Xiao W Jiang Z Chen S Guo D. Single-cell characterization of Malignant phenotypes and microenvironment alteration in retinoblastoma. Cell Death Dis (2022) 13:438. doi: 10.1038/s41419-022-04904-8 35523772 PMC9076657

[25] Obradovic A Chowdhury N Haake SM Ager C Wang V Vlahos L. Single-cell protein activity analysis identifies recurrence-associated renal tumor macrophages. Cell (2021) 184:2988–3005.e16. doi: 10.1016/j.cell.2021.04.038 34019793 PMC8479759

[26] Zhao S Wang L Ding W Ye B Cheng C Shao J. Crosstalk of disulfidptosis-related subtypes, establishment of a prognostic signature and immune infiltration characteristics in bladder cancer based on a machine learning survival framework. Front Endocrinol (Lausanne) (2023) 14:1180404. doi: 10.3389/fendo.2023.1180404 37152941 PMC10154596

[27] Lee HO Hong Y Etlioglu HE Cho YB Pomella V Van den Bosch B. Lineage-dependent gene expression programs influence the immune landscape of colorectal cancer. Nat Genet (2020) 52:594–603. doi: 10.1038/s41588-020-0636-z 32451460

[28] Qiu X Mao Q Tang Y Wang L Chawla R Pliner HA. Reversed graph embedding resolves complex single-cell trajectories. Nat Methods (2017) 14:979–82. doi: 10.1038/nmeth.4402 PMC5764547 28825705

[29] Puram SV Tirosh I Parikh AS Patel AP Yizhak K Gillespie S. Single-cell transcriptomic analysis of primary and metastatic tumor ecosystems in head and neck cancer. Cell (2017) 171:1611–1624.e24. doi: 10.1016/j.cell.2017.10.044 29198524 PMC5878932

[30] Jin S Li R Chen MY Yu C Tang LQ Liu YM. Single-cell transcriptomic analysis defines the interplay between tumor cells, viral infection, and the microenvironment in nasopharyngeal carcinoma. Cell Res (2020) 30:950–65. doi: 10.1038/s41422-020-00402-8 PMC7784966 32901110

[31] Gao Y Wang H Chen S An R Chu Y Li G. Single-cell N(6)-methyladenosine regulator patterns guide intercellular communication of tumor microenvironment that contribute to colorectal cancer progression and immunotherapy. J Transl Med (2022) 20:197. doi: 10.1186/s12967-022-03395-7 35509079 PMC9066909

[32] Wu Y Yang S Ma J Chen Z Song G Rao D. Spatiotemporal immune landscape of colorectal cancer liver metastasis at single-cell level. Cancer Discovery (2022) 12:134–53. doi: 10.1158/2159-8290.Cd-21-0316 34417225

[33] Aibar S González-Blas CB Moerman T Huynh-Thu VA Imrichova H Hulselmans G. SCENIC: single-cell regulatory network inference and clustering. Nat Methods (2017) 14:1083–6. doi: 10.1038/nmeth.4463 PMC5937676 28991892

[34] Jin S Guerrero-Juarez CF Zhang L Chang I Ramos R Kuan CH. Inference and analysis of cell-cell communication using CellChat. Nat Commun (2021) 12:1088. doi: 10.1038/s41467-021-21246-9 33597522 PMC7889871

[35] Jiang P Gu S Pan D Fu J Sahu A Hu X. Signatures of T cell dysfunction and exclusion predict cancer immunotherapy response. Nat Med (2018) 24:1550–8. doi: 10.1038/s41591-018-0136-1 PMC6487502 30127393

[36] Lambrechts D Wauters E Boeckx B Aibar S Nittner D Burton O. Phenotype molding of stromal cells in the lung tumor microenvironment. Nat Med (2018) 24:1277–89. doi: 10.1038/s41591-018-0096-5 29988129

[37] Davidson G Helleux A Vano YA Lindner V Fattori A Cerciat M. Mesenchymal-like tumor cells and myofibroblastic cancer-associated fibroblasts are associated with progression and immunotherapy response of clear cell renal cell carcinoma. Cancer Res (2023) 83:2952–69. doi: 10.1158/0008-5472.Can-22-3034 37335139

[38] Zhang Q He Y Luo N Patel SJ Han Y Gao R. Landscape and dynamics of single immune cells in hepatocellular carcinoma. Cell (2019) 179:829–845.e20. doi: 10.1016/j.cell.2019.10.003 31675496

[39] Bayik D Lathia JD. Cancer stem cell-immune cell crosstalk in tumour progression. Nat Rev Cancer (2021) 21:526–36. doi: 10.1038/s41568-021-00366-w PMC8740903 34103704

[40] Chen Z Zhou L Liu L Hou Y Xiong M Yang Y. Single-cell RNA sequencing highlights the role of inflammatory cancer-associated fibroblasts in bladder urothelial carcinoma. Nat Commun (2020) 11:5077. doi: 10.1038/s41467-020-18916-5 33033240 PMC7545162

[41] Galbo PM Jr. Zang X Zheng D. Molecular features of cancer-associated fibroblast subtypes and their implication on cancer pathogenesis, prognosis, and immunotherapy resistance. Clin Cancer Res (2021) 27:2636–47. doi: 10.1158/1078-0432.Ccr-20-4226 PMC8102353 33622705

[42] Patel SA Hirosue S Rodrigues P Vojtasova E Richardson EK Ge J. The renal lineage factor PAX8 controls oncogenic signalling in kidney cancer. Nature (2022) 606:999–1006. doi: 10.1038/s41586-022-04809-8 35676472 PMC9242860

[43] Kanno T Kamba T Yamasaki T Shibasaki N Saito R Terada N. JunB promotes cell invasion and angiogenesis in VHL-defective renal cell carcinoma. Oncogene (2012) 31:3098–110. doi: 10.1038/onc.2011.475 22020339

[44] van Vlerken-Ysla L Tyurina YY Kagan VE Gabrilovich DI. Functional states of myeloid cells in cancer. Cancer Cell (2023) 41:490–504. doi: 10.1016/j.ccell.2023.02.009 36868224 PMC10023509

[45] Shi R Tang YQ Miao H. Metabolism in tumor microenvironment: Implications for cancer immunotherapy. MedComm (2020) (2020) 1:47–68. doi: 10.1002/mco2.6 34766109 PMC8489668

[46] Zhu J Yamane H Paul WE. Differentiation of effector CD4 T cell populations (*). Annu Rev Immunol (2010) 28:445–89. doi: 10.1146/annurev-immunol-030409-101212 PMC3502616 20192806

[47] St Paul M Ohashi PS. The roles of CD8(+) T cell subsets in antitumor immunity. Trends Cell Biol (2020) 30:695–704. doi: 10.1016/j.tcb.2020.06.003 32624246

[48] Chen Z Yang X Bi G Liang J Hu Z Zhao M. Ligand-receptor interaction atlas within and between tumor cells and T cells in lung adenocarcinoma. Int J Biol Sci (2020) 16:2205–19. doi: 10.7150/ijbs.42080 PMC7294944 32549766

[49] Wang T Guo K Zhang D Wang H Yin J Cui H. Disulfidptosis classification of hepatocellular carcinoma reveals correlation with clinical prognosis and immune profile. Int Immunopharmacol (2023) 120:110368. doi: 10.1016/j.intimp.2023.110368 37247499

[50] Liu F Yuan D Liu X Zhuo S Liu X Sheng H. A demonstration based on multi-omics transcriptome sequencing data revealed disulfidptosis heterogeneity within the tumor microenvironment of esophageal squamous cell carcinoma. Discovery Oncol (2023) 14:96. doi: 10.1007/s12672-023-00711-5 PMC10260728 37306828

[51] Xiao L Yin W Chen X Zhang X Zhang C Yu Z. A disulfidptosis-related lncRNA index predicting prognosis and the tumor microenvironment in colorectal cancer. Sci Rep (2023) 13:20135. doi: 10.1038/s41598-023-47472-3 37978247 PMC10656577

[52] Feng Z Zhao Q Ding Y Xu Y Sun X Chen Q. Identification a unique disulfidptosis classification regarding prognosis and immune landscapes in thyroid carcinoma and providing therapeutic strategies. J Cancer Res Clin Oncol (2023) 149:11157–70. doi: 10.1007/s00432-023-05006-4 PMC11796729 37347261

[53] de Visser KE Joyce JA. The evolving tumor microenvironment: From cancer initiation to metastatic outgrowth. Cancer Cell (2023) 41:374–403. doi: 10.1016/j.ccell.2023.02.016 36917948

[54] Tlsty TD Coussens LM. Tumor stroma and regulation of cancer development. Annu Rev Pathol (2006) 1:119–50. doi: 10.1146/annurev.pathol.1.110304.100224 18039110

[55] Raskov H Orhan A Christensen JP Gögenur I. Cytotoxic CD8(+) T cells in cancer and cancer immunotherapy. Br J Cancer (2021) 124:359–67. doi: 10.1038/s41416-020-01048-4 PMC7853123 32929195

[56] Li Y Li G Zhang J Wu X Chen X. The dual roles of human γδ T cells: Anti-tumor or tumor-promoting. Front Immunol (2020) 11:619954. doi: 10.3389/fimmu.2020.619954 33664732 PMC7921733

[57] Kalluri R. The biology and function of fibroblasts in cancer. Nat Rev Cancer (2016) 16:582–98. doi: 10.1038/nrc.2016.73 27550820

[58] Hanahan D Coussens LM. Accessories to the crime: functions of cells recruited to the tumor microenvironment. Cancer Cell (2012) 21:309–22. doi: 10.1016/j.ccr.2012.02.022 22439926

[59] Mhaidly R Mechta-Grigoriou F. Fibroblast heterogeneity in tumor micro-environment: Role in immunosuppression and new therapies. Semin Immunol (2020) 48:101417. doi: 10.1016/j.smim.2020.101417 33077325

[60] Pei L Liu Y Liu L Gao S Gao X Feng Y. Roles of cancer-associated fibroblasts (CAFs) in anti- PD-1/PD-L1 immunotherapy for solid cancers. Mol Cancer (2023) 22:29. doi: 10.1186/s12943-023-01731-z 36759842 PMC9912573

[61] Sharon Y Alon L Glanz S Servais C Erez N. Isolation of normal and cancer-associated fibroblasts from fresh tissues by Fluorescence Activated Cell Sorting (FACS). J Vis Exp (2013) 71:e4425. doi: 10.3791/4425 PMC3582516 23354290

[62] Fiori ME Di Franco S Villanova L Bianca P Stassi G De Maria R. Cancer-associated fibroblasts as abettors of tumor progression at the crossroads of EMT and therapy resistance. Mol Cancer (2019) 18:70. doi: 10.1186/s12943-019-0994-2 30927908 PMC6441236

[63] Yoshimatsu Y Wakabayashi I Kimuro S Takahashi N Takahashi K Kobayashi M. TNF-α enhances TGF-β-induced endothelial-to-mesenchymal transition via TGF-β signal augmentation. Cancer Sci (2020) 111:2385–99. doi: 10.1111/cas.14455 PMC7385392 32385953

[64] Zeisberg EM Potenta S Xie L Zeisberg M Kalluri R. Discovery of endothelial to mesenchymal transition as a source for carcinoma-associated fibroblasts. Cancer Res (2007) 67:10123–8. doi: 10.1158/0008-5472.Can-07-3127 17974953

[65] Mishra PJ Mishra PJ Glod JW Banerjee D. Mesenchymal stem cells: flip side of the coin. Cancer Res (2009) 69:1255–8. doi: 10.1158/0008-5472.Can-08-3562 19208837

[66] Mao X Xu J Wang W Liang C Hua J Liu J. Crosstalk between cancer-associated fibroblasts and immune cells in the tumor microenvironment: new findings and future perspectives. Mol Cancer (2021) 20:131. doi: 10.1186/s12943-021-01428-1 34635121 PMC8504100

[67] McAndrews KM Chen Y Darpolor JK Zheng X Yang S Carstens JL. Identification of functional heterogeneity of carcinoma-associated fibroblasts with distinct IL6-mediated therapy resistance in pancreatic cancer. Cancer Discovery (2022) 12:1580–97. doi: 10.1158/2159-8290.Cd-20-1484 PMC9399904 35348629

[68] Li YX Zhu XX Wu X Li JH Ni XH Li SJ. ACLP promotes activation of cancer-associated fibroblasts and tumor metastasis via ACLP-PPARγ-ACLP feedback loop in pancreatic cancer. Cancer Lett (2022) 544:215802. doi: 10.1016/j.canlet.2022.215802 35732215

[69] Li YY Tao YW Gao S Li P Zheng JM Zhang SE. Cancer-associated fibroblasts contribute to oral cancer cells proliferation and metastasis *via* exosome-mediated paracrine miR-34a-5p. EBioMedicine (2018) 36:209–20. doi: 10.1016/j.ebiom.2018.09.006 PMC6197737 30243489

[70] Huang Y Chen Z Lu T Bi G Li M Liang J. HIF-1α switches the functionality of TGF-β signaling via changing the partners of smads to drive glucose metabolic reprogramming in non-small cell lung cancer. J Exp Clin Cancer Res (2021) 40:398. doi: 10.1186/s13046-021-02188-y 34930376 PMC8690885

[71] Zhan S Li J Wang T Ge W. Quantitative proteomics analysis of sporadic medullary thyroid cancer reveals FN1 as a potential novel candidate prognostic biomarker. Oncologist (2018) 23:1415–25. doi: 10.1634/theoncologist.2017-0399 PMC6292558 29739896

[72] Shao C Cheng C Shao Q Chen B. Identification and validation of biglycan as prognosis and therapy markers for patients with stomach adenocarcinoma. Int J Gen Med (2021) 14:3497–509. doi: 10.2147/ijgm.S321641 PMC8290488 34295178

[73] Winkler J Abisoye-Ogunniyan A Metcalf KJ Werb Z. Concepts of extracellular matrix remodelling in tumour progression and metastasis. Nat Commun (2020) 11:5120. doi: 10.1038/s41467-020-18794-x 33037194 PMC7547708

[74] Tan B Shi X Zhang J Qin J Zhang N Ren H. Inhibition of rspo-lgr4 facilitates checkpoint blockade therapy by switching macrophage polarization. Cancer Res (2018) 78:4929–42. doi: 10.1158/0008-5472.Can-18-0152 29967265

[75] Comito G Giannoni E Segura CP Barcellos-de-Souza P Raspollini MR Baroni G. Cancer-associated fibroblasts and M2-polarized macrophages synergize during prostate carcinoma progression. Oncogene (2014) 33:2423–31. doi: 10.1038/onc.2013.191 23728338

[76] Christofides A Strauss L Yeo A Cao C Charest A Boussiotis VA. The complex role of tumor-infiltrating macrophages. Nat Immunol (2022) 23:1148–56. doi: 10.1038/s41590-022-01267-2 PMC10754321 35879449

[77] Mao C Huang C Hu Z Qu S. Transcription factor CASZ1 increases an oncogenic transcriptional process in tumorigenesis and progression of glioma cells. MedComm (2020) (2022) 3:e182. doi: 10.1002/mco2.182 36276925 PMC9583698

[78] Ioannou I Chatziantoniou A Drenios C Christodoulou P Kourti M Zaravinos A. Signatures of co-deregulated genes and their transcriptional regulators in kidney cancers. Int J Mol Sci (2023) 24:6577. doi: 10.3390/ijms24076577 37047552 PMC10094846

[79] Zhang C Zhang J Liang F Guo H Gao S Yang F. Innate immune checkpoint Siglec10 in cancers: mining of comprehensive omics data and validation in patient samples. Front Med (2022) 16:596–609. doi: 10.1007/s11684-021-0868-z 35075579

[80] Doan H Parsons A Devkumar S Selvarajah J Miralles F Carroll VA. HIF-mediated suppression of DEPTOR confers resistance to mTOR kinase inhibition in renal cancer. iScience (2019) 21:509–20. doi: 10.1016/j.isci.2019.10.047 PMC6849413 31710966

[81] Song Z Li Z Han W Zhu C Lou N Li X. Low DAPK1 expression correlates with poor prognosis and sunitinib resistance in clear cell renal cell carcinoma. Aging (Albany NY) (2020) 13:1842–58. doi: 10.18632/aging.103638 PMC7880360 33201837

[82] Dai J Lu Y Wang J Yang L Han Y Wang Y. A four-gene signature predicts survival in clear-cell renal-cell carcinoma. Oncotarget (2016) 7:82712–26. doi: 10.18632/oncotarget.12631 PMC5347726 27779101

[83] Lu J Qian C Ji Y Bao Q Lu B. Gene signature associated with bromodomain genes predicts the prognosis of kidney renal clear cell carcinoma. Front Genet (2021) 12:643935. doi: 10.3389/fgene.2021.643935 34149798 PMC8206647

[84] Gao K Zhang F Chen K Li W Guan YB Xu ML. Expression patterns and prognostic value of RUNX genes in kidney cancer. Sci Rep (2021) 11:14934. doi: 10.1038/s41598-021-94294-2 34294773 PMC8298387

[85] Cowman SJ Fuja DG Liu XD Tidwell RSS Kandula N Sirohi D. Macrophage HIF-1α Is an independent prognostic indicator in kidney cancer. Clin Cancer Res (2020) 26:4970–82. doi: 10.1158/1078-0432.Ccr-19-3890 PMC7968518 32586940

[86] Qu S Huang C Zhu T Wang K Zhang H Wang L. OLFML3, as a potential predictor of prognosis and therapeutic target for glioma, is closely related to immune cell infiltration. VIEW (2023) 4:20220052. doi: 10.1002/VIW.20220052

[87] Hu Z Qu S. EVA1C is a potential prognostic biomarker and correlated with immune infiltration levels in WHO grade II/III glioma. Front Immunol (2021) 12:683572. doi: 10.3389/fimmu.2021.683572 34267752 PMC8277382

[88] Kurebayashi Y Ojima H Tsujikawa H Kubota N Maehara J Abe Y. Landscape of immune microenvironment in hepatocellular carcinoma and its additional impact on histological and molecular classification. Hepatology (2018) 68:1025–41. doi: 10.1002/hep.29904 29603348

[89] Chen Y Jin C Cui J Diao Y Wang R Xu R. Single-cell sequencing and bulk RNA data reveal the tumor microenvironment infiltration characteristics of disulfidptosis related genes in breast cancer. J Cancer Res Clin Oncol (2023) 149:12145–64. doi: 10.1007/s00432-023-05109-y PMC11796854 37428249

[90] Qu S Qiu O Huang J Liu J Wang H. Upregulation of hsa-miR-196a-5p is associated with MIR196A2 methylation and affects the Malignant biological behaviors of glioma. Genomics (2021) 113:1001–10. doi: 10.1016/j.ygeno.2021.02.012 33636314

[91] Qu S Qiu O Hu Z. The prognostic factors and nomogram for patients with high-grade gliomas. Fundam Res (2021) 1:824–8. doi: 10.1016/j.fmre.2021.07.005

[92] Zhang S-W Wang H Ding X-H Xiao Y-L Shao Z-M You C. Bidirectional crosstalk between therapeutic cancer vaccines and the tumor microenvironment: Beyond tumor antigens. Fundam Res (2022) 3:1005–24. doi: 10.1016/j.fmre.2022.03.009 PMC11197801 38933006

[93] Yang N Li H Cao C Zhao L Song X Wang W. Tumor microenvironment-activated theranostic nanoreactor for NIR-II Photoacoustic imaging-guided tumor-specific photothermal therapy. Fundam Res (2022). doi: 10.1016/j.fmre.2022.04.021 PMC11197737 38933846

[94] Guan L Chen J Tian Z Zhu M Bian Y Zhu Y. Mesoporous organosilica nanoparticles: Degradation strategies and application in tumor therapy. VIEW (2021) 2:20200117. doi: 10.1002/VIW.20200117

[95] Protti MP De Monte L Di Lullo G. Tumor antigen-specific CD4+ T cells in cancer immunity: from antigen identification to tumor prognosis and development of therapeutic strategies. Tissue Antigens (2014) 83:237–46. doi: 10.1111/tan.12329 24641502

[96] Seung E Xing Z Wu L Rao E Cortez-Retamozo V Ospina B. A trispecific antibody targeting HER2 and T cells inhibits breast cancer growth via CD4 cells. Nature (2022) 603:328–34. doi: 10.1038/s41586-022-04439-0 35197632

[97] Oh DY Kwek SS Raju SS Li T McCarthy E Chow E. Intratumoral CD4(+) T cells mediate anti-tumor cytotoxicity in human bladder cancer. Cell (2020) 181:1612–1625.e13. doi: 10.1016/j.cell.2020.05.017 32497499 PMC7321885

[98] Peña-Romero AC Orenes-Piñero E. Dual effect of immune cells within tumour microenvironment: Pro- and anti-tumour effects and their triggers. Cancers (Basel) (2022) 14:1681. doi: 10.3390/cancers14071681 35406451 PMC8996887

[99] Maj T Wang W Crespo J Zhang H Wang W Wei S. Oxidative stress controls regulatory T cell apoptosis and suppressor activity and PD-L1-blockade resistance in tumor. Nat Immunol (2017) 18:1332–41. doi: 10.1038/ni.3868 PMC5770150 29083399

[100] Jiang YQ Wang ZX Zhong M Shen LJ Han X Zou X. Investigating mechanisms of response or resistance to immune checkpoint inhibitors by analyzing cell-cell communications in tumors before and after programmed cell death-1 (PD-1) targeted therapy: An integrative analysis using single-cell RNA and bulk-RNA sequencing data. Oncoimmunology (2021) 10:1908010. doi: 10.1080/2162402x.2021.1908010 33868792 PMC8023241

