# Supplementary material for: Single-cell disulfidptosis regulator patterns guide intercellular communication of tumor microenvironment that contribute to kidney renal clear cell carcinoma progression and immunotherapy
Source: Front Immunol. 2024 Jan 16;15:1288240. doi: 10.3389/fimmu.2024.1288240 (PMC10824999; doi:10.3389/fimmu.2024.1288240)
Supplement: Supplementary file 1 [file Table_1.docx]

**TableS1 The Geneset lists using in the study**

| **Gene Set names** | **Types** | **Genes** | | | | | | |
| --- | --- | --- | --- | --- | --- | --- | --- | --- |
| **Disulfidptosis genes** |  | INF2 | CD2AP | ACTN4 | PDLIM1 | DSTN | IQGAP1 | CAPZB |
|  |  | ACTB | MYH10 | MYL6 | MYH9 | TLN1 | FLNA | FLNB |
|  |  | SLC7A11 | SLC3A2 | RPN1 | NCKAP1 | NUBPL | NDUFA11 | LRPPRC |
|  |  | OXSM | NDUFS1 | GYS1 |  |  |  |  |
| **CCR CAF**  **genes** | **pan-myCAF** | ADIRF | ACTA2 | MYH11 | TAGLN | SPARCL1 | MCAM | A2M |
|  |  | PPP1R14A | CRIP2 | ADAMTS1 | CSRP2 | NDUFA4L2 | TPM1 | MAP1B |
|  |  | RGS5 | MEF2C | CALM2 | APOLD1 | OAZ2 | MGST3 | ISYNA1 |
|  |  | NDRG2 | ID3 | RGS16 | CYB5R3 | CRYAB | OLFML2A | TIMP3 |
|  |  | MYLK | IGFBP7 | CRIP1 | TINAGL1 | TPM2 | PTP4A3 | CPE |
|  |  | FRZB | PRKCDBP | CSRP1 | CAV1 | ADAMTS4 | GJA4 |  |
|  |  | CPM | PGF | GUCY1B3 | UBA2 | YIF1A | PHLDA1 |  |
|  |  | GUCY1A3 | FILIP1 | FAM13C | NDUFS4 | ITGB1 | KCNE4 |  |
|  | **pan-dCAF** | COL1A1 | THBS2 | CTHRC1 | COL3A1 | LUM | COL1A2 | LGALS1 |
|  |  | COL12A1 | MMP2 | INHBA | TNFAIP6 | VCAN | ADAM12 | THY1 |
|  |  | EMP1 | ANGPTL2 | RARRES2 | SULF1 | IGFBP3 | COL8A1 | GREM1 |
|  |  | P4HA3 | CRABP2 | TPM4 | LOXL1 | CPXM1 | COL6A1 | ASPN |
|  |  | YIF1A | SNAI2 | C1S | TMEM176B | CTGF | MORF4L2 | UAP1 |
|  |  | LMNA | CYP1B1 | MGP | ANGPTL4 | TIMP1 | SERPINF1 | ITGB1 |
|  |  | COL5A1 | POSTN | SERPINE1 | LOXL2 | COL11A1 | CTSK | TGFBI |
|  |  | FN1 | STEAP1 | SPON2 | PLAUR | SPHK1 | LOX | HTRA3 |
|  |  | DCN | ITGA5 | ANTXR1 | RIN2 | TMEM119 | TNFRSF12A | C1R |
|  |  | PDLIM4 | ITGA11 | PRSS23 | COL6A2 | SFRP2 | PLAU |  |
|  | **pan-iCAF** | CFD | GPC3 | C3 | ADH1B | IGF1 | EFEMP1 | PODN |
|  |  | PLA2G2A | DPT | WISP2 | CCDC80 | SFRP2 | PTGDS | DCN |
|  |  | FHL2 | ELN | KLF4 | TMEM176B | SERPINF1 | FHL1 | GPX3 |
|  |  | RARRES1 | CYR61 | IGFBP5 | FBLN1 | MGST1 | MFAP4 | CTGF |
|  |  | SEPP1 | CXCL12 | ABI3BP | GSN | TMEM176A | FIBIN | C1R |
|  |  | MGP | C1S | IGFBP6 | CYP1B1 | CST3 | SLC40A1 | SFRP4 |
|  | **pan-pCAF** | NUSAP1 | DIAPH3 | LOXL2 | ADAM12 | LOX | POSTN | THY1 |
|  |  | COL1A1 | COL8A1 | COL6A1 | COL6A2 | CD248 | FN1 | COL12A1 |
|  |  | CTHRC1 | COL5A1 | LOXL1 |  |  |  |  |
|  | **pan-iCAF-2** | IER3 | CXCL2 | ICAM1 | TNFAIP2 | NFKBIA | NR4A1 | CCL2 |
|  |  | GEM | NR4A3 | APOD | SAT1 | UAP1 | OGN | GFPT2 |
|  |  | ABI3BP | GADD45B | DUSP1 | RARRES1 | CST3 | ABL2 | EGR1 |
|  |  | SOD2 | FOSB | PIM1 | ZFP36 | CLU | C3 | CYP1B1 |
|  |  | JUNB | THBS1 | CDKN1A |  |  |  |  |
| **T cell**  **Marker genes** | **Co-inhibitors** | ADORA2A | BTLA | BTN2A2 | BTN3A1 | BTN3A2 | BTNL2 | C10orf54 |
|  |  | CSF1R | HAVCR2 | IDO1 | IL10 | IL10RB | KDR | KIR2DL1 |
|  |  | SLAMF7 | TGFB1 | TIGIT | VRCN1 | VTCN1 | CD247 | CTLA4 |
|  |  | CD160 | CD244 | CD274 | CD276 | CD48 | CD96 |  |
|  |  | KIR2DL2 | KIR2DL3 | LAG3 | LAIR1 | LGALS9 | PVRL2 |  |
|  |  | PDCD1 | PDCD1LG2 |  |  |  |  |  |
|  | **Co-stimuiations** | BTNL8 | CD226 | CD27 | CD28 | CD40 | CD58 | CD70 |
|  |  | SLAMF1 | TMIGD2 | TNFRSF13B | TNFRSF13C | TNFRSF14 | TNFRSF4 | TNFRSF8 |
|  |  | TNFSF8 | TNFSF9 | ENTPD1 | NT5E | ICOS | TNFSF4 | TNFSF15 |
|  |  | CD80 | CD86 | EGFR | HAVCR1 | TNFSF18 | ICOSLG | TNFSF13B |
|  |  | TNFRSF9 | TNFSF13 |  |  |  |  |  |
|  | **T-function Genes** | CD3E | CD4 | CD8B | FOXP3 | GZMB | PRF1 | TBX21 |
|  |  | IL2RA | IKZF2 |  |  |  |  |  |
| **T Function Score gene**  **list** | **Teffectscore** | CD8A | CXCL10 | CXCL9 | GZMA | GZMB | IFNG | PRF1 |
|  |  | TBX21 |  |  |  |  |  |  |
|  | **Cytotoxicscore** | CST7 | GZMA | GZMB | IFNG | NKG7 | PRF1 |  |
|  | **Exhaustionscore** | CTLA4 | HAVCR2 | LAG3 | PDCD1 | TIGIT |  |  |
|  | **Tevasionscore** | B2M | IFNGR1 | JAK2 | SOCS1 | TAP1 | TAP2 | TAPBP |
|  |  | AGO2 | AHSA1 | ANAPC15 | ARF6 | ATG14 | ATG7 | ATG9A |
|  |  | PDCD6IP | PDSS2 | PI4KB | PIGK | PIGT | PKN2 | PPP1CA |
|  |  | PDIA3 | PIGS | PIGU | PPP2R2A | PSMB8 | RB1CC1 | RBM15 |
|  |  | IFNAR1 | IFNAR2 | IKBKB | INO80 | IPPK | IRF9 | JAGN1 |
|  |  | GPI1 | IFNGR2 | IKBKG | IRF1 | JAK1 | OTUD5 | PTPN2 |
|  |  | BRPF1 | CAD | CALR | CEP55 | CHMP5 | COX6C | CREBBP |
|  |  | RCE1 | RGP1 | RIC1 | S100PBP | SARNP | SETD1A | SETDB1 |
|  |  | TMEM127 | TNFRSF1B | UBE2G2 | UBE2N | VDAC2 | VPS29 | VPS35 |
|  |  | MED16 | MED23 | MED24 | MGAT1 | MTA2 | N6AMT1 | NCBP1 |
|  |  | ATG12 | ATG3 | ATG5 | BECN1 | CHIC2 | DNTTIP1 | EMC8 |
|  |  | DPH5 | EIF3H | EMC2 | EMC3 | EMC4 | EMC6 | F8A |
|  |  | TBK1 | TFRC | TGFBR2 | TMEM208 | TRADD | TRPM7 | UBR5 |
|  |  | MAP3K7 | MEN1 | MOGS | NDUFA13 | NXT1 | OTULIN | H2-K1 |
|  |  | VPS16 | WDR7 | WDR83 | WWP2 | YAP1 | ZC3H3 | ACTB |
|  |  | ADAR | ATG10 | ATG101 | CFLAR | FITM2 | GPAA1 | PCED1B |
|  |  | ATP13A1 | ATXN7L3 | BC003331 | BCL2L1 | BOLA3 | BRAT1 | IST1 |
|  |  | PPP1R8 | PPP2R3C | PRKCSH | PSMB9 | PSMG1 | PTAR1 | GLS |
|  |  | RIC8 | RNF31 | SCAF4 | SMG7 | TAB1 | TAB2 | VPS13A |
|  |  | JMJD6 | KAT6A | KLF16 | KMT2A | LIPT2 | MAPK1 | HSPA13 |
|  |  | RBCK1 | STAT1 | STAT2 | TNFAIP3 | TNFRSF1A | TRAF2 | ACAD9 |
|  |  | CUL3 | DCP1A | DET1 | DICER1 | DNAJC13 | DOT1L | PCBP2 |
|  |  | SLC25A32 | SPCS1 | SRRT | SRSF7 | STOML2 | SUSD6 | HDAC1 |
|  |  | VPS4B | WIPI2 | ZCCHC14 | FAM58B | FAS | FNTB | GALE |
|  |  | NDUFAF6 | NPLOC4 | NUP188 | UFC1 | UFL1 | USP7 | UXS1 |
|  |  | ERAP1 | ERP44 | FADD | HCFC2 | HDGFRP2 | HEXIM1 | HIRA |
