# Supplementary material for: Single-cell disulfidptosis regulator patterns guide intercellular communication of tumor microenvironment that contribute to kidney renal clear cell carcinoma progression and immunotherapy
Source: Front Immunol. 2024 Jan 16;15:1288240. doi: 10.3389/fimmu.2024.1288240 (PMC10824999; doi:10.3389/fimmu.2024.1288240)
Supplement: Supplementary file 2 [file Table_2.docx]

**TableS2 KEGG analysis for Each disulfidptosis-mediated Fibroblasts subClusters**

| **ID** | **Description** | **Gene**  **Ratio** | **Bg**  **Ratio** | **pvalue** | **p.adjust** | **qvalue** | **Count** | **log.p.adjust** | **Celltype** |
| --- | --- | --- | --- | --- | --- | --- | --- | --- | --- |
| hsa04270 | Vascular smooth muscle contraction | 4/11 | 134/8577 | 1.73E-05 | 0.00089789 | 0.000581629 | 4 | 7.015463143 | Fibroblasts_C1 |
| hsa04814 | Motor proteins | 3/11 | 193/8577 | 0.00161997 | 0.024352002 | 0.015774576 | 3 | 3.715141213 | Fibroblasts_C1 |
| hsa04510 | Focal adhesion | 3/11 | 203/8577 | 0.001873231 | 0.024352002 | 0.015774576 | 3 | 3.715141213 | Fibroblasts_C1 |
| hsa05415 | Diabetic cardiomyopathy | 3/11 | 203/8577 | 0.001873231 | 0.024352002 | 0.015774576 | 3 | 3.715141213 | Fibroblasts_C1 |
| hsa04810 | Regulation of actin cytoskeleton | 3/11 | 229/8577 | 0.00264462 | 0.027504046 | 0.017816386 | 3 | 3.593422153 | Fibroblasts_C1 |
| hsa03320 | PPAR signaling pathway | 2/11 | 75/8577 | 0.003943167 | 0.034174117 | 0.02213708 | 2 | 3.376286733 | Fibroblasts_C1 |
| hsa04260 | Cardiac muscle contraction | 2/11 | 87/8577 | 0.005271358 | 0.039018022 | 0.025274832 | 2 | 3.243731644 | Fibroblasts_C1 |
| hsa04520 | Adherens junction | 2/11 | 93/8577 | 0.006002773 | 0.039018022 | 0.025274832 | 2 | 3.243731644 | Fibroblasts_C1 |
| hsa04510 | Focal adhesion | 3/7 | 203/8577 | 0.000426234 | 0.014065708 | 0.010319339 | 3 | 4.264015521 | Fibroblasts_C2 |
| hsa05165 | Human papillomavirus infection | 3/7 | 331/8577 | 0.00177552 | 0.017729956 | 0.013007623 | 3 | 4.032499642 | Fibroblasts_C2 |
| hsa04512 | ECM-receptor interaction | 2/7 | 89/8577 | 0.002161509 | 0.017729956 | 0.013007623 | 2 | 4.032499642 | Fibroblasts_C2 |
| hsa04151 | PI3K-Akt signaling pathway | 3/7 | 359/8577 | 0.002244269 | 0.017729956 | 0.013007623 | 3 | 4.032499642 | Fibroblasts_C2 |
| hsa04933 | AGE-RAGE signaling pathway in diabetic complications | 2/7 | 100/8577 | 0.002720543 | 0.017729956 | 0.013007623 | 2 | 4.032499642 | Fibroblasts_C2 |
| hsa04066 | HIF-1 signaling pathway | 2/7 | 109/8577 | 0.003223628 | 0.017729956 | 0.013007623 | 2 | 4.032499642 | Fibroblasts_C2 |
| hsa04970 | Salivary secretion | 1/2 | 93/8577 | 0.021569585 | 0.021800238 | NA | 1 | 3.825834379 | Fibroblasts_C4 |
| hsa04657 | IL-17 signaling pathway | 1/2 | 94/8577 | 0.021800238 | 0.021800238 | NA | 1 | 3.825834379 | Fibroblasts_C4 |
