# Supplementary material for: Single-cell disulfidptosis regulator patterns guide intercellular communication of tumor microenvironment that contribute to kidney renal clear cell carcinoma progression and immunotherapy
Source: Front Immunol. 2024 Jan 16;15:1288240. doi: 10.3389/fimmu.2024.1288240 (PMC10824999; doi:10.3389/fimmu.2024.1288240)
Supplement: Supplementary file 3 [file Table_3.docx]

**TableS2 Gene list of disulfidptosis-related genes in each NMF sub clusters**

| **Disulfidptosis celltype** | **Disulfidptosis related gene** | **Disulfidptosis celltype** | **Disulfidptosis related gene** | **Disulfidptosis celltype** | **Disulfidptosis related gene** |
| --- | --- | --- | --- | --- | --- |
| Fibroblast_C1 | ACTN4 | Macrophage_C1 | FLNA | CD4+ T cells_C1 | DSTN |
| Fibroblast_C1 | NDUFA11 | Macrophage_C1 | S100A6 | CD4+ T cells_C1 | TLN1 |
| Fibroblast_C1 | TPM1 | Macrophage_C1 | S100A10 | CD4+ T cells_C1 | CAPZB |
| Fibroblast_C1 | ACTA2 | Macrophage_C1 | S100A4 | CD4+ T cells_C1 | FLNA |
| Fibroblast_C1 | MYLK | Macrophage_C1 | CD52 | CD4+ T cells_C1 | KLRD1 |
| Fibroblast_C1 | MYL9 | Macrophage_C1 | SLC2A3 | CD4+ T cells_C1 | NKG7 |
| Fibroblast_C1 | TAGLN | Macrophage_C1 | FCN1 | CD4+ T cells_C1 | GZMB |
| Fibroblast_C1 | INPP4B | Macrophage_C1 | MXD1 | CD4+ T cells_C1 | GNLY |
| Fibroblast_C1 | COX4I2 | Macrophage_C1 | VIM | CD4+ T cells_C1 | CCL4 |
| Fibroblast_C1 | CD36 | Macrophage_C1 | TIMP1 | CD4+ T cells_C1 | SLC3A2 |
| Fibroblast_C1 | PPP1R14A | Macrophage_C1 | VCAN | CD4+ T cells_C1 | HSPE1 |
| Fibroblast_C1 | FABP4 | Macrophage_C1 | ATP1B3 | CD4+ T cells_C1 | TXNIP |
| Fibroblast_C2 | PDLIM1 | Macrophage_C1 | ANXA2 | CD4+ T cells_C1 | IER3 |
| Fibroblast_C2 | LOX | Macrophage_C1 | EZR | CD4+ T cells_C2 | FLNA |
| Fibroblast_C2 | TNC | Macrophage_C1 | LMNA | CD4+ T cells_C2 | IQGAP1 |
| Fibroblast_C2 | ABI3BP | Macrophage_C1 | EMP3 | CD4+ T cells_C2 | AHNAK |
| Fibroblast_C2 | PLOD2 | Macrophage_C1 | SH3BGRL3 | CD4+ T cells_C2 | GOLGA8A |
| Fibroblast_C2 | SERPINE1 | Macrophage_C1 | ANXA1 | CD4+ T cells_C2 | PRDM1 |
| Fibroblast_C2 | VEGFA | Macrophage_C1 | EREG | CD4+ T cells_C2 | TTN |
| Fibroblast_C2 | S100A10 | Macrophage_C1 | RPS26 | CD4+ T cells_C2 | MYH9 |
| Fibroblast_C2 | TGFBI | Macrophage_C1 | INSIG1 | CD4+ T cells_C2 | BRD2 |
| Fibroblast_C2 | COL6A3 | Macrophage_C1 | RGCC | CD4+ T cells_C2 | DNAJB12 |
| Fibroblast_C2 | ANXA2 | Macrophage_C1 | SAMSN1 | CD4+ T cells_C2 | STAT2 |
| Fibroblast_C2 | XIST | Macrophage_C1 | THBS1 | CD4+ T cells_C2 | PICALM |
| Fibroblast_C3 | NDUFA11 | Macrophage_C1 | CLEC10A | CD4+ T cells_C2 | MT-ND4L |
| Fibroblast_C3 | TIMP1 | Macrophage_C1 | FN1 | CD4+ T cells_C2 | DCP1A |
| Fibroblast_C4 | S100A9 | Macrophage_C2 | DSTN | CD4+ T cells_C2 | ST3GAL1 |
| Fibroblast_C4 | LYZ | Macrophage_C3 | CCL3 | CD4+ T cells_C2 | PFKFB3 |
| Fibroblast_C4 | XIST | Macrophage_C3 | CMTM2 | CD4+ T cells_C2 | SYNE2 |
| Fibroblast_C5 | CAPZB | Macrophage_C3 | S100A12 | CD4+ T cells_C2 | NFKBIZ |
| CD8+ T cells_C2 | FLNA | Macrophage_C3 | MYL6 | CD4+ T cells_C2 | KIAA1551 |
| CD8+ T cells_C2 | SLC3A2 | Macrophage_C3 | S100P | CD4+ T cells_C2 | VCP |
| CD8+ T cells_C2 | CAPZB | Macrophage_C3 | FCGR3B | CD4+ T cells_C2 | JMJD6 |
| CD8+ T cells_C2 | TLN1 | Macrophage_C3 | AQP9 | CD4+ T cells_C2 | ATRX |
| CD8+ T cells_C2 | MYH9 | Macrophage_C3 | PREX1 | CD4+ T cells_C2 | NEAT1 |
| CD8+ T cells_C2 | MALAT1 | Macrophage_C3 | S100A8 | CD4+ T cells_C2 | CHTOP |
| CD8+ T cells_C2 | MT-ND2 | Macrophage_C3 | VPS37B | CD4+ T cells_C2 | 7SK.2 |
| CD8+ T cells_C2 | HIST1H3D | Macrophage_C3 | DAZAP2 | CD4+ T cells_C2 | SUGP2 |
| CD8+ T cells_C2 | TTN | Macrophage_C3 | ARHGDIA | CD4+ T cells_C2 | MYL6 |
| CD8+ T cells_C2 | CHD2 | Macrophage_C3 | ACTB | CD4+ T cells_C2 | AP4B1 |
| CD8+ T cells_C2 | SYNE2 | Macrophage_C3 | RB1CC1 | CD4+ T cells_C2 | ATF4 |
| CD8+ T cells_C2 | EGR3 | Macrophage_C3 | TXNIP | CD4+ T cells_C2 | RBBP6 |
| CD8+ T cells_C2 | SCLT1 | Macrophage_C3 | RAB7A | CD4+ T cells_C2 | KIAA1683 |
| CD8+ T cells_C2 | RMRP | Macrophage_C3 | BCL2A1 | CD4+ T cells_C2 | SLC38A2 |
| CD8+ T cells_C2 | CCDC93 | Macrophage_C3 | PTPLAD2 | CD4+ T cells_C2 | DDX39B |
| CD8+ T cells_C2 | APOLD1 | Macrophage_C3 | LAMTOR5 | CD4+ T cells_C2 | ELK4 |
| CD8+ T cells_C2 | IGFBP7 | Macrophage_C3 | GNB2 | CD4+ T cells_C2 | FUS |
| CD8+ T cells_C2 | GTPBP1 | Macrophage_C3 | MBOAT7 | CD4+ T cells_C2 | PRF1 |
| CD8+ T cells_C2 | IQGAP1 | Macrophage_C3 | LMAN2 | CD4+ T cells_C2 | ATP1B1 |
| CD8+ T cells_C2 | AGAP1 | Macrophage_C3 | SLA | CD4+ T cells_C2 | TLN1 |
| CD8+ T cells_C2 | NBEAL2 | Macrophage_C3 | TSPAN14 | CD4+ T cells_C2 | HNRNPA2B1 |
| CD8+ T cells_C2 | KDM5D | Macrophage_C3 | SEMA4A | CD4+ T cells_C2 | FEZ2 |
| CD8+ T cells_C2 | MACF1 | Macrophage_C3 | BAX | CD4+ T cells_C2 | BAZ1B |
| CD8+ T cells_C2 | MIAT | Macrophage_C3 | VAPA | CD4+ T cells_C2 | JUN |
| CD8+ T cells_C2 | PLEKHA1 | Macrophage_C3 | PDCD4 | CD4+ T cells_C2 | KLF13 |
| CD8+ T cells_C2 | P2RY8 | Macrophage_C3 | PRR13 | CD4+ T cells_C2 | RFNG |
| CD8+ T cells_C2 | FAM129A | Macrophage_C3 | BANF1 | CD4+ T cells_C2 | PPP1R10 |
| CD8+ T cells_C2 | TTTY15 | Macrophage_C3 | EVI2B | CD4+ T cells_C2 | CTA-29F11.1 |
| CD8+ T cells_C2 | CCND2 | Macrophage_C3 | CYB5A | CD4+ T cells_C2 | TRA2B |
| CD8+ T cells_C2 | FAM13B | Macrophage_C3 | SKAP2 | CD4+ T cells_C2 | ARRDC1 |
| CD8+ T cells_C2 | MYCBP2 | Macrophage_C3 | VCP | CD4+ T cells_C2 | DDX27 |
| CD8+ T cells_C2 | NPIPB5 | Macrophage_C3 | TGM2 | CD4+ T cells_C2 | ATP1A1 |
| CD8+ T cells_C2 | GLS | Macrophage_C3 | NRD1 | CD4+ T cells_C2 | NFKB2 |
| CD8+ T cells_C2 | KPNB1 | Macrophage_C3 | WAC | CD4+ T cells_C2 | CBL |
| CD8+ T cells_C2 | SLC38A1 | Macrophage_C3 | S100A9 | CD4+ T cells_C2 | MACF1 |
| CD8+ T cells_C2 | MED17 | Macrophage_C3 | CACUL1 | CD4+ T cells_C2 | MT-ATP6 |
| CD8+ T cells_C2 | JOSD1 | Macrophage_C3 | LSMEM1 | CD4+ T cells_C2 | MT-CO2 |
| CD8+ T cells_C2 | PHF1 | Macrophage_C3 | FOXO3 | CD4+ T cells_C2 | MALAT1 |
| CD8+ T cells_C2 | EPAS1 | Macrophage_C3 | ABI1 | CD4+ T cells_C2 | MT-ND2 |
| CD8+ T cells_C2 | ZC3HAV1 | Macrophage_C3 | PEBP1 | CD4+ T cells_C2 | MT-ND1 |
| CD8+ T cells_C2 | ERCC6L2 | Macrophage_C3 | MRPL51 | CD4+ T cells_C2 | MT-CO1 |
| CD8+ T cells_C2 | ACIN1 | Macrophage_C3 | IFNGR2 | CD4+ T cells_C2 | MT-CO3 |
| CD8+ T cells_C2 | SPTAN1 | Macrophage_C3 | HNRNPK | CD4+ T cells_C2 | MT-ND3 |
| CD8+ T cells_C2 | AHNAK | Macrophage_C3 | VPS4B | CD4+ T cells_C2 | CAPN15 |
| CD8+ T cells_C2 | RP11-138A9.1 | Macrophage_C3 | NDUFC1 | CD4+ T cells_C2 | MT-ND4 |
| CD8+ T cells_C2 | MIDN | Macrophage_C3 | TRIM69 | CD4+ T cells_C2 | SFPQ |
| CD8+ T cells_C2 | PPP2R5C | Macrophage_C3 | CAP1 | CD4+ T cells_C2 | HNRNPH1 |
| CD8+ T cells_C2 | AC016831.7 | Macrophage_C3 | ADIPOR1 | CD4+ T cells_C2 | CLSTN1 |
| CD8+ T cells_C2 | SERINC1 | Macrophage_C3 | CARD8 | CD4+ T cells_C2 | SFT2D2 |
| CD8+ T cells_C2 | SLC38A2 | Macrophage_C3 | MYO5A | CD4+ T cells_C2 | MBTD1 |
| CD8+ T cells_C2 | PTPN22 | Macrophage_C3 | BCL6 | CD4+ T cells_C2 | ASNS |
| CD8+ T cells_C2 | MTRNR2L12 | Macrophage_C3 | CLTA | CD4+ T cells_C2 | RP11-773D16.1 |
| CD8+ T cells_C2 | ADIRF | Macrophage_C4 | CAPZB | CD4+ T cells_C2 | TTC13 |
| CD8+ T cells_C2 | PCBP1-AS1 | Macrophage_C4 | HLA-DQA2 | CD4+ T cells_C2 | PNISR |
| CD8+ T cells_C2 | RLIM | Macrophage_C4 | TXNIP | CD4+ T cells_C2 | SRSF7 |
| CD8+ T cells_C2 | IFRD1 | Macrophage_C4 | APOE | CD4+ T cells_C2 | RGS5 |
| CD8+ T cells_C2 | CREBRF | Macrophage_C4 | TMEM176B | CD4+ T cells_C2 | PSMD5 |
| CD8+ T cells_C2 | PDE4B | Macrophage_C4 | GADD45G | CD4+ T cells_C2 | PACS2 |
| CD8+ T cells_C2 | NEAT1 | Macrophage_C4 | PDK4 | CD4+ T cells_C2 | GLIPR1 |
| CD8+ T cells_C2 | MT-CO1 | Macrophage_C4 | APOC1 | CD4+ T cells_C2 | RBM12 |
| CD8+ T cells_C2 | MT-CO2 | Macrophage_C4 | C1QA | CD4+ T cells_C2 | PPP2R5C |
| CD8+ T cells_C2 | MT-ND1 | Macrophage_C4 | C1QB | CD4+ T cells_C2 | PDE7A |
| CD8+ T cells_C2 | MT-ND4 | Macrophage_C4 | CTSD | CD4+ T cells_C2 | C1orf63 |
| CD8+ T cells_C2 | MT-ATP6 | Macrophage_C4 | MS4A4A | CD4+ T cells_C2 | MT-ND5 |
| CD8+ T cells_C2 | MT-ND3 | Macrophage_C4 | DAB2 | CD4+ T cells_C2 | LSMEM1 |
| CD8+ T cells_C2 | MT-ND5 | Macrophage_C4 | C1QC | CD4+ T cells_C2 | TMEM2 |
| CD8+ T cells_C2 | TMEM2 | Macrophage_C4 | FOLR2 | CD4+ T cells_C2 | NR2C1 |
| CD8+ T cells_C2 | MT-CYB | Macrophage_C4 | JUN | CD4+ T cells_C3 | ACTN4 |
| CD8+ T cells_C2 | TSPYL2 | Macrophage_C4 | C3 | CD4+ T cells_C3 | TLN1 |
| CD8+ T cells_C2 | HNRNPA2B1 | Macrophage_C4 | KLF2 | CD4+ T cells_C3 | CAPZB |
| CD8+ T cells_C2 | HNRNPH1 | Macrophage_C4 | MAFB | CD4+ T cells_C3 | ACTN4 |
| CD8+ T cells_C2 | RUNX3 | Macrophage_C4 | SEPP1 | CD4+ T cells_C3 | DSTN |
| CD8+ T cells_C2 | FUS | Macrophage_C4 | HMOX1 | CD4+ T cells_C3 | S100A4 |
| CD8+ T cells_C2 | SRSF5 | Macrophage_C4 | ID2 | CD4+ T cells_C3 | GZMB |
| CD8+ T cells_C2 | RP11-166P13.4 | Macrophage_C4 | CXCL10 | CD4+ T cells_C3 | GNLY |
| CD8+ T cells_C2 | NEU1 | Macrophage_C4 | NDUFA11 | CD4+ T cells_C3 | CCL20 |
| CD8+ T cells_C2 | RNU12 | Macrophage_C4 | FILIP1L | CD4+ T cells_C3 | SLC3A2 |
| CD8+ T cells_C3 | ACTN4 | Macrophage_C4 | ACTB | CD4+ T cells_C3 | FLNA |
| CD8+ T cells_C3 | FLNA | Macrophage_C4 | NDUFA12 | CD4+ T cells_C3 | IQGAP1 |
| CD8+ T cells_C3 | SLC3A2 | Macrophage_C4 | ARL5A | CD4+ T cells_C3 | MACF1 |
| CD8+ T cells_C3 | TLN1 | Macrophage_C4 | ANXA7 | CD4+ T cells_C3 | SLFN5 |
| CD8+ T cells_C3 | IQGAP1 | Macrophage_C4 | VPS28 | CD4+ T cells_C3 | PRF1 |
| CD8+ T cells_C3 | DNAJA4 | Macrophage_C4 | RB1 | CD4+ T cells_C3 | SYNE2 |
| CD8+ T cells_C3 | MS4A6A | Macrophage_C4 | PSMB6 | CD4+ T cells_C3 | XIST |
| CD8+ T cells_C3 | MYH9 | Macrophage_C4 | ACP1 | CD4+ T cells_C3 | FAM111A |
| CD8+ T cells_C3 | CAPZB | Macrophage_C4 | PURB | CD4+ T cells_C3 | PLEKHO2 |
| CD8+ T cells_C3 | IGFBP7 | Macrophage_C4 | ENSA | CD4+ T cells_C3 | GPR183 |
| CD8+ T cells_C3 | ERCC6L2 | Macrophage_C4 | SNRPB2 | CD4+ T cells_C3 | 7SK.2 |
| CD8+ T cells_C3 | PHF1 | Macrophage_C4 | PTBP1 | CD4+ T cells_C3 | JUN |
| CD8+ T cells_C3 | SBDS | Macrophage_C4 | SRP14 | CD4+ T cells_C3 | NKTR |
| CD8+ T cells_C3 | DUSP1 | Macrophage_C4 | RAN | CD4+ T cells_C3 | MTRNR2L2 |
| CD8+ T cells_C3 | ARL4D | Macrophage_C4 | HN1 | CD4+ T cells_C3 | MYH9 |
| CD8+ T cells_C3 | ADIRF | Macrophage_C4 | G3BP2 | CD4+ T cells_C3 | ZFR |
| CD8+ T cells_C3 | TNRC6A | Macrophage_C4 | SCO2 | CD4+ T cells_C3 | NPM1 |
| CD8+ T cells_C3 | RBM4 | Macrophage_C4 | SNRPD2 | CD4+ T cells_C3 | CCR7 |
| CD8+ T cells_C3 | IL7R | Macrophage_C4 | ZFAND2A | CD4+ T cells_C3 | FTH1 |
| CD8+ T cells_C3 | TXNIP | Macrophage_C4 | SNRPD3 | CD4+ T cells_C3 | IRS2 |
| CD8+ T cells_C3 | RGS5 | Macrophage_C4 | TXNL1 | CD4+ T cells_C3 | CHMP1B |
| CD8+ T cells_C3 | FGD5-AS1 | Macrophage_C4 | RAC2 | CD4+ T cells_C3 | PRDX1 |
| CD8+ T cells_C3 | CXCL13 | Macrophage_C4 | EEF2 | CD4+ T cells_C3 | GZMK |
| CD8+ T cells_C3 | SPG11 | Macrophage_C4 | YWHAE | CD4+ T cells_C3 | RICTOR |
| CD8+ T cells_C3 | RP11-640M9.1 | Macrophage_C4 | ATP5I | CD4+ T cells_C3 | PTGDR |
| CD8+ T cells_C3 | ID2 | Macrophage_C4 | VPS29 | CD4+ T cells_C3 | PPP6R2 |
| CD8+ T cells_C3 | ETV3 | Macrophage_C4 | DHX36 | CD4+ T cells_C3 | PSMB2 |
| CD8+ T cells_C3 | RGS2 | Macrophage_C4 | FNBP1 | CD4+ T cells_C3 | UHMK1 |
| CD8+ T cells_C3 | MYADM | Macrophage_C4 | JTB | CD4+ T cells_C3 | DNAJA1 |
| CD8+ T cells_C3 | ATAD2B | Macrophage_C4 | BHLHE40 | CD4+ T cells_C3 | HSPE1 |
| CD8+ T cells_C3 | KAT5 | Macrophage_C4 | ARFGAP3 | CD4+ T cells_C3 | MRPL18 |
| CD8+ T cells_C3 | APOL1 | Macrophage_C4 | NLRP3 | CD4+ T cells_C3 | EGR1 |
| CD8+ T cells_C3 | NR4A3 | Macrophage_C4 | ZNF638 | CD4+ T cells_C3 | GOLGB1 |
| CD8+ T cells_C3 | ATP6V1H | Macrophage_C4 | TUBB | CD4+ T cells_C3 | LMNA |
| CD8+ T cells_C3 | C9orf89 | Macrophage_C4 | ANP32B | CD4+ T cells_C3 | B4GALT1 |
| CD8+ T cells_C3 | CCL4 | Macrophage_C4 | FKBP4 | CD4+ T cells_C3 | ZNF644 |
| CD8+ T cells_C3 | BFAR | Macrophage_C4 | RAP2B | CD4+ T cells_C3 | SCML4 |
| CD8+ T cells_C3 | TSC22D1 | Macrophage_C4 | TES | CD4+ T cells_C3 | FOS |
| CD8+ T cells_C3 | MALAT1 | Macrophage_C4 | SNAP23 | CD4+ T cells_C3 | ABCF1 |
| CD8+ T cells_C3 | MT-ND5 | NK cells_C1 | DSTN | CD4+ T cells_C3 | SLAMF6 |
| CD8+ T cells_C3 | MT-ND2 | NK cells_C1 | SLC3A2 | CD4+ T cells_C3 | ACTR8 |
| CD8+ T cells_C3 | MT-CO1 | NK cells_C1 | HSPA6 | CD4+ T cells_C3 | CKLF |
| CD8+ T cells_C3 | MT-ATP6 | NK cells_C1 | ZFP36 | CD4+ T cells_C3 | ADAM10 |
| CD8+ T cells_C3 | SLC16A4 | NK cells_C1 | TNFAIP3 | CD4+ T cells_C3 | RP11-796E2.4 |
| CD8+ T cells_C3 | TMEM175 | NK cells_C1 | AC016831.7 | CD4+ T cells_C3 | HSPH1 |
| CD8+ T cells_C3 | FAM122B | NK cells_C1 | WDR74 | CD4+ T cells_C3 | SQSTM1 |
| CD8+ T cells_C3 | RPS6KA3 | NK cells_C1 | TLN1 | CD4+ T cells_C3 | HSP90AA1 |
| CD8+ T cells_C3 | POLR2F | NK cells_C2 | TLN1 | CD4+ T cells_C3 | DNAJB1 |
| CD8+ T cells_C3 | FCGR3A | NK cells_C2 | KIN | CD4+ T cells_C3 | HSPA1A |
| CD8+ T cells_C3 | PPP2CA | NK cells_C2 | BRMS1 | CD4+ T cells_C3 | HSPD1 |
| CD8+ T cells_C3 | PEBP1 | NK cells_C2 | TNK2 | CD4+ T cells_C3 | LRRC8A |
| CD8+ T cells_C3 | PPDPF | NK cells_C2 | PILRB | CD4+ T cells_C3 | SYNC |
| CD8+ T cells_C3 | TTC39B | NK cells_C2 | ZNF292 | CD4+ T cells_C3 | MAP1LC3A |
| CD8+ T cells_C3 | CCDC6 | NK cells_C2 | CRTC3 | CD4+ T cells_C3 | DNAJA4 |
| CD8+ T cells_C3 | CNDP2 | NK cells_C2 | EGR1 | CD4+ T cells_C3 | GS1-251I9.4 |
| CD8+ T cells_C3 | TNIP3 | NK cells_C2 | ARHGAP26 | CD4+ T cells_C3 | RALGDS |
| CD8+ T cells_C3 | EIF3J | NK cells_C2 | NSUN6 | CD4+ T cells_C3 | MT-ND5 |
| CD8+ T cells_C3 | YTHDF2 | NK cells_C2 | IQGAP1 | CD4+ T cells_C3 | MALAT1 |
| CD8+ T cells_C3 | MT-ATP8 | NK cells_C2 | HERC4 | CD4+ T cells_C3 | MT-ND4 |
| CD8+ T cells_C3 | ZNF302 | NK cells_C2 | GPRIN3 | CD4+ T cells_C3 | MT-ND1 |
| CD8+ T cells_C3 | HIST1H3D | NK cells_C2 | HP1BP3 | CD4+ T cells_C3 | MT-CO1 |
| CD8+ T cells_C3 | MT-ND3 | NK cells_C2 | KIAA1551 | CD4+ T cells_C3 | MT-ATP6 |
| CD8+ T cells_C3 | MT-ND1 | NK cells_C2 | MACF1 | CD4+ T cells_C3 | MT-CO3 |
| CD8+ T cells_C3 | MT-CO2 | NK cells_C2 | THEMIS2 | CD4+ T cells_C3 | MT-ND2 |
| CD8+ T cells_C3 | MT-ND4 | NK cells_C2 | MYH9 | CD4+ T cells_C3 | MT-ND3 |
| CD8+ T cells_C3 | NEAT1 | NK cells_C2 | KRT81 | CD4+ T cells_C3 | PPP1R10 |
| CD8+ T cells_C3 | MT-CO3 | NK cells_C2 | LINC00324 | CD4+ T cells_C3 | ELMO2 |
| CD8+ T cells_C3 | KLRC1 | NK cells_C2 | 7SK.2 | CD4+ T cells_C3 | MT-CYB |
| CD8+ T cells_C3 | STK4 | NK cells_C2 | CYP20A1 | CD4+ T cells_C3 | MTRNR2L12 |
| CD8+ T cells_C3 | SYNE2 | NK cells_C2 | DPP9 | CD4+ T cells_C3 | IER2 |
| CD8+ T cells_C3 | MT-ND4L | NK cells_C2 | ZNF638 | CD4+ T cells_C3 | MTRNR2L8 |
| CD8+ T cells_C3 | MTRNR2L12 | NK cells_C2 | FOXO1 | CD4+ T cells_C3 | HNRNPA2B1 |
| CD8+ T cells_C3 | C22orf46 | NK cells_C2 | PSD4 | CD4+ T cells_C3 | C1orf63 |
| CD8+ T cells_C3 | MT-CYB | NK cells_C2 | PPP1R10 | CD4+ T cells_C3 | FAM129A |
| CD8+ T cells_C3 | SLC17A5 | NK cells_C3 | IQGAP1 | CD4+ T cells_C3 | IGFBP7 |
| CD8+ T cells_C3 | ZNF680 | NK cells_C3 | FLNA | CD4+ T cells_C3 | RGS5 |
| CD8+ T cells_C3 | DGKD | NK cells_C3 | STT3A | CD4+ T cells_C3 | RNF213 |
| CD8+ T cells_C3 | HES1 | NK cells_C3 | SOS2 | CD4+ T cells_C3 | TRRAP |
| CD8+ T cells_C3 | MTRNR2L2 | NK cells_C3 | CERS2 | CD4+ T cells_C3 | TULP4 |
| CD8+ T cells_C3 | HEBP1 | NK cells_C3 | SRGAP3 | CD4+ T cells_C3 | FAM126B |
| CD8+ T cells_C3 | HSD17B4 | NK cells_C3 | FRMD4B | CD4+ T cells_C3 | KIAA0196 |
| CD8+ T cells_C3 | DNAJB4 | NK cells_C3 | GZMK | CD4+ T cells_C3 | ZKSCAN1 |
| CD8+ T cells_C3 | HSPA1A | NK cells_C3 | ABHD3 | CD4+ T cells_C3 | HNRNPH1 |
| CD8+ T cells_C3 | JUN | NK cells_C3 | TRANK1 | CD4+ T cells_C3 | SMG1 |
| CD8+ T cells_C3 | CDKN1C | NK cells_C4 | NDUFA11 | CD4+ T cells_C3 | CYTH4 |
| CD8+ T cells_C3 | MORF4L2 | NK cells_C4 | PTGDS | CD4+ T cells_C3 | PHF10 |
| CD8+ T cells_C3 | DCUN1D4 | NK cells_C4 | CAPZB | CD4+ T cells_C3 | GBP5 |
| CD8+ T cells_C3 | HSPA1B | NK cells_C4 | ACTN4 | CD4+ T cells_C3 | TPM2 |
| CD8+ T cells_C3 | FAM179B | NK cells_C4 | SLC3A2 | CD4+ T cells_C3 | LINC00426 |
| CD8+ T cells_C3 | TLE4 | NK cells_C4 | MYH9 | CD4+ T cells_C3 | SLC35E2B |
| CD8+ T cells_C3 | TAF7 | NK cells_C4 | DSTN | CD4+ T cells_C3 | ANKRD13D |
| CD8+ T cells_C3 | RNF185 | NK cells_C4 | DUSP1 | CD4+ T cells_C3 | ANAPC4 |
| CD8+ T cells_C3 | BCL6 | NK cells_C4 | ZNF259 | CD4+ T cells_C3 | SON |
| CD8+ T cells_C3 | LINC00944 | NK cells_C4 | CHCHD7 | CD4+ T cells_C3 | CREG1 |
| CD8+ T cells_C3 | DIS3L | NK cells_C4 | FLNA | CD4+ T cells_C3 | EIF4A2 |
| CD8+ T cells_C3 | CCDC130 | NK cells_C4 | RP6-99M1.2 | CD4+ T cells_C3 | TM9SF2 |
| CD8+ T cells_C3 | DGKH | NK cells_C4 | NEAT1 | CD4+ T cells_C3 | PYCR2 |
| CD8+ T cells_C3 | RING1 | NK cells_C4 | LRCH3 | CD4+ T cells_C3 | VMP1 |
| CD8+ T cells_C3 | CD4 | NK cells_C4 | TMEM2 | CD4+ T cells_C3 | IPO9 |
| CD8+ T cells_C3 | CD2AP | NK cells_C4 | MSL2 | CD4+ T cells_C3 | TEFM |
| CD8+ T cells_C3 | CHST7 | NK cells_C4 | ZBED4 | CD4+ T cells_C3 | JAK3 |
| CD8+ T cells_C3 | HNRNPH1 | NK cells_C4 | ARIH1 | CD4+ T cells_C3 | ZNF493 |
| CD8+ T cells_C3 | RANBP2 | NK cells_C4 | ATP2A2 | CD4+ T cells_C3 | VPS13A |
| CD8+ T cells_C3 | TTN | NK cells_C4 | FBRSL1 | CD4+ T cells_C3 | TRA2B |
| CD8+ T cells_C3 | PPP1R13B | NK cells_C4 | HNRNPH1 | CD4+ T cells_C3 | ITGA4 |
| CD8+ T cells_C3 | 7SK.2 | NK cells_C4 | PDXDC1 | CD4+ T cells_C3 | FGD3 |
| CD8+ T cells_C3 | AGPAT6 | NK cells_C4 | UHMK1 | CD4+ T cells_C3 | BPTF |
| CD8+ T cells_C3 | TNK2 | NK cells_C4 | MPPE1 | CD4+ T cells_C3 | FAM78A |
| CD8+ T cells_C3 | RBM39 | NK cells_C4 | ZBTB40 | CD4+ T cells_C3 | NEU1 |
| CD8+ T cells_C3 | TYMP | NK cells_C4 | SLC38A2 | CD4+ T cells_C3 | ZBTB7A |
| CD8+ T cells_C3 | ABCA7 | NK cells_C4 | IVNS1ABP | CD4+ T cells_C3 | DNAJC10 |
| CD8+ T cells_C3 | KEAP1 | NK cells_C4 | APLP2 | CD4+ T cells_C3 | HOXB2 |
| CD8+ T cells_C3 | USP20 | NK cells_C4 | USP34 | CD4+ T cells_C3 | HLA-F |
| CD8+ T cells_C3 | KIAA0930 | NK cells_C4 | PSME4 | CD4+ T cells_C3 | TGFBR2 |
| CD8+ T cells_C3 | SNX25 | NK cells_C4 | RLIM | CD4+ T cells_C3 | RP11-66N24.4 |
| CD8+ T cells_C3 | RPGR | NK cells_C4 | PCF11 | CD4+ T cells_C3 | NEAT1 |
| CD8+ T cells_C3 | CDK5RAP1 | NK cells_C4 | SYNE1 | CD4+ T cells_C3 | PGPEP1 |
| CD8+ T cells_C3 | MAP3K10 | NK cells_C4 | PCMTD2 | CD4+ T cells_C3 | PRR3 |
| CD8+ T cells_C3 | RABGEF1 | NK cells_C4 | RNF103 | CD4+ T cells_C3 | POLR3E |
| CD8+ T cells_C3 | PAFAH2 | NK cells_C4 | GK5 | CD4+ T cells_C3 | KRIT1 |
| CD8+ T cells_C3 | ACBD4 | NK cells_C4 | PCNX | CD4+ T cells_C3 | ZMAT1 |
| CD8+ T cells_C3 | TMEM38B | NK cells_C4 | CREBRF | CD4+ T cells_C3 | MT-CO2 |
| CD8+ T cells_C3 | MBNL1 | NK cells_C4 | SGK1 | CD4+ T cells_C3 | PNISR |
| CD8+ T cells_C3 | ACIN1 | NK cells_C4 | SLC2A3 | CD4+ T cells_C3 | COPS7A |
| CD8+ T cells_C3 | ACAP3 | NK cells_C4 | FBXO33 | CD4+ T cells_C3 | C5orf51 |
| CD8+ T cells_C3 | CCDC125 | NK cells_C4 | KLF4 | CD4+ T cells_C3 | RAB5B |
| CD8+ T cells_C3 | POC1B | NK cells_C4 | TNFAIP3 | CD4+ T cells_C3 | SMCHD1 |
| CD8+ T cells_C3 | KANSL1 | NK cells_C4 | CMTR2 | CD4+ T cells_C4 | MALAT1 |
| CD8+ T cells_C3 | B3GALTL | NK cells_C4 | PPP1R13B | CD4+ T cells_C4 | MT-ND2 |
| CD8+ T cells_C3 | VPS33A | NK cells_C4 | PDE4B | CD4+ T cells_C4 | MT-CO1 |
| CD8+ T cells_C3 | C20orf194 | NK cells_C4 | SKIL | CD4+ T cells_C4 | MTRNR2L12 |
| CD8+ T cells_C3 | PIGG | NK cells_C4 | ELK3 | CD4+ T cells_C4 | NEAT1 |
| CD8+ T cells_C3 | TNFAIP3 | NK cells_C4 | AOAH | CD4+ T cells_C4 | 7SK.2 |
| CD8+ T cells_C3 | SLC31A1 | NK cells_C4 | GLCCI1 | CD4+ T cells_C4 | MT-ND5 |
| CD8+ T cells_C3 | NSUN3 | NK cells_C4 | WIPF1 | CD4+ T cells_C4 | SRSF7 |
| CD8+ T cells_C3 | KLHL42 | NK cells_C4 | RANBP6 | CD4+ T cells_C4 | FUS |
| CD8+ T cells_C3 | PRR4 | NK cells_C4 | ARHGAP35 | CD4+ T cells_C4 | RN7SL1 |
| CD8+ T cells_C3 | AC016586.1 | NK cells_C4 | HACL1 | CD4+ T cells_C4 | RP11-307C12.13 |
| CD8+ T cells_C3 | NAP1L2 | NK cells_C4 | UBR4 | CD4+ T cells_C4 | MALAT1 |
| CD8+ T cells_C3 | RP11-2B6.2 | NK cells_C4 | IQGAP1 | CD4+ T cells_C4 | AC068620.1 |
| CD8+ T cells_C3 | ZMAT1 | NK cells_C4 | STRBP | CD4+ T cells_C4 | MT-ATP6 |
| CD8+ T cells_C3 | WDR44 | NK cells_C4 | NSF | CD4+ T cells_C4 | MT-CO3 |
| CD8+ T cells_C3 | CACTIN | NK cells_C4 | RP11-796E2.4 | CD4+ T cells_C4 | MT-ND3 |
| CD8+ T cells_C3 | ZNF821 | NK cells_C4 | CDK16 | CD4+ T cells_C4 | RP11-575L7.8 |
| CD8+ T cells_C3 | ITGA2 | NK cells_C4 | CDK19 | CD4+ T cells_C4 | MT-ND1 |
| CD8+ T cells_C3 | RP11-325F22.2 | NK cells_C4 | RICTOR | CD4+ T cells_C4 | UAP1L1 |
| CD8+ T cells_C3 | KIAA0100 | NK cells_C4 | ETS1 | CD4+ T cells_C4 | PARP14 |
| CD8+ T cells_C3 | PRIMPOL | NK cells_C4 | LRRFIP2 | CD4+ T cells_C4 | MT-CYB |
| CD8+ T cells_C3 | KIF9 | NK cells_C4 | CEP290 | CD4+ T cells_C4 | MT-CO2 |
| CD8+ T cells_C3 | CCDC136 | NK cells_C4 | DDX27 | CD4+ T cells_C4 | GSN |
| CD8+ T cells_C3 | HKR1 | NK cells_C4 | MTRNR2L2 | CD4+ T cells_C4 | RP1-187B23.1 |
| CD8+ T cells_C3 | AC079341.1 | NK cells_C4 | ITGA1 | CD4+ T cells_C4 | APC |
| CD8+ T cells_C3 | SLC37A3 | NK cells_C4 | DIDO1 | CD4+ T cells_C4 | PIK3AP1 |
| CD8+ T cells_C3 | LZTR1 | NK cells_C4 | ATXN7 | CD4+ T cells_C4 | SLC10A7 |
| CD8+ T cells_C3 | MEX3B | NK cells_C4 | TAF8 | CD4+ T cells_C4 | TMEM135 |
| CD8+ T cells_C3 | NXPE3 | NK cells_C4 | PDLIM1 | CD4+ T cells_C4 | MT-ND4 |
| CD8+ T cells_C3 | SCML1 | NK cells_C4 | ADRBK1 | CD4+ T cells_C4 | FN1 |
| CD8+ T cells_C3 | ZNF655 | NK cells_C4 | SRGAP3 | CD4+ T cells_C4 | MT-ND4L |
| CD8+ T cells_C3 | NUP155 | NK cells_C4 | MIR29A | CD4+ T cells_C4 | ARHGAP21 |
| CD8+ T cells_C3 | MYCBP2 | NK cells_C5 | XCL1 | CD4+ T cells_C4 | WDR3 |
| CD8+ T cells_C3 | SLC35A2 | NK cells_C5 | 7SK.2 | CD4+ T cells_C4 | MTRNR2L8 |
| CD8+ T cells_C3 | MALSU1 | NK cells_C5 | CD44 | CD4+ T cells_C5 | CAPZB |
| CD8+ T cells_C3 | TRIP6 | NK cells_C5 | HIST2H2AA3 | CD4+ T cells_C5 | SLC3A2 |
| CD8+ T cells_C3 | CTNNAL1 | NK cells_C5 | STK17A | CD4+ T cells_C5 | CCR7 |
| CD8+ T cells_C3 | PELI1 | NK cells_C5 | MALAT1 | CD4+ T cells_C5 | MYL6 |
| CD8+ T cells_C3 | NCBP1 | NK cells_C5 | MT-ND1 | CD4+ T cells_C5 | MALAT1 |
| CD8+ T cells_C3 | DUSP3 | NK cells_C5 | MT-ND4L | CD4+ T cells_C5 | MT-ND2 |
| CD8+ T cells_C3 | HAUS3 | NK cells_C5 | MT-ND2 | CD4+ T cells_C5 | MT-ATP6 |
| CD8+ T cells_C3 | ZNF571-AS1 | NK cells_C5 | MT-ND3 | CD4+ T cells_C5 | MT-ND1 |
| CD8+ T cells_C3 | AC004951.6 | NK cells_C5 | TUBGCP6 | CD4+ T cells_C5 | MT-CO1 |
| CD8+ T cells_C3 | LMOD3 | NK cells_C5 | TRIM24 | CD4+ T cells_C5 | MT-CO3 |
| CD8+ T cells_C3 | ANKRD36 | NK cells_C5 | CCNL2 | CD4+ T cells_C5 | MT-ND4 |
| CD8+ T cells_C3 | BET1L | NK cells_C5 | ENOPH1 | CD4+ T cells_C5 | 7SK.2 |
| CD8+ T cells_C3 | CCZ1B | B cells_C1 | NDUFA11 | CD4+ T cells_C5 | MT-ND3 |
| CD8+ T cells_C3 | CRYAB | B cells_C1 | FKBP11 | CD4+ T cells_C5 | MT-CO2 |
| CD8+ T cells_C3 | CKB | B cells_C1 | DERL3 | CD4+ T cells_C5 | MT-CYB |
| CD8+ T cells_C3 | SON | B cells_C1 | PRDX4 | CD4+ T cells_C5 | MT-ND5 |
| CD8+ T cells_C4 | ADIRF | B cells_C1 | MZB1 | CD4+ T cells_C5 | MTRNR2L12 |
| CD8+ T cells_C4 | MGP | B cells_C1 | SPAG4 | CD4+ T cells_C5 | MTRNR2L8 |
| CD8+ T cells_C4 | MALAT1 | B cells_C1 | SDF2L1 | CD4+ T cells_C5 | SRSF7 |
| CD8+ T cells_C4 | MT-ND2 | B cells_C1 | FKBP2 | CD4+ T cells_C5 | NPEPPS |
| CD8+ T cells_C4 | MT-CO1 | B cells_C1 | NUCB2 | CD4+ T cells_C5 | MT-ND4L |
| CD8+ T cells_C4 | MT-ND1 | B cells_C1 | XBP1 | CD4+ T cells_C5 | MTRNR2L2 |
| CD8+ T cells_C4 | NEAT1 | B cells_C1 | SSR4 | CD4+ T cells_C5 | MCTP2 |
| CD8+ T cells_C4 | TIMP3 | B cells_C1 | SEC11C | Monocyte_C1 | RPN1 |
| CD8+ T cells_C4 | FUS | B cells_C1 | ERLEC1 | Monocyte_C1 | SLC3A2 |
| CD8+ T cells_C4 | MT-CO2 | B cells_C1 | TNFRSF17 | Monocyte_C1 | CD163 |
| CD8+ T cells_C4 | MYL6 | B cells_C1 | ITM2C | Monocyte_C1 | XIST |
| CD8+ T cells_C4 | MT-ATP6 | B cells_C1 | JSRP1 | Monocyte_C1 | VEGFA |
| CD8+ T cells_C4 | MT-ND3 | B cells_C1 | SDC1 | Monocyte_C1 | RANBP2 |
| CD8+ T cells_C4 | MT-ND4 | B cells_C1 | VIMP | Monocyte_C1 | CCND2 |
| CD8+ T cells_C4 | MT-ND5 | B cells_C1 | ARSA | Monocyte_C1 | LMNA |
| CD8+ T cells_C4 | MT-CYB | B cells_C1 | FNDC3B | Monocyte_C1 | RB1 |
| CD8+ T cells_C4 | MTRNR2L12 | B cells_C1 | DSTN | Monocyte_C1 | THBS1 |
| CD8+ T cells_C4 | RGS5 | B cells_C1 | CD38 | Monocyte_C2 | ACTN4 |
| CD8+ T cells_C4 | MT-CO3 | B cells_C1 | C19orf10 | Monocyte_C2 | S100A8 |
| CD8+ T cells_C4 | HNRNPA2B1 | B cells_C1 | HDLBP | Monocyte_C3 | HSPA1A |
| CD8+ T cells_C4 | NKTR | B cells_C1 | CREB3L2 | Monocyte_C3 | IER5 |
| CD8+ T cells_C4 | CCDC64 | B cells_C1 | PPIB | Monocyte_C3 | DNAJB1 |
| CD8+ T cells_C4 | SNTB1 | B cells_C1 | HM13 | Monocyte_C3 | BAG3 |
| CD8+ T cells_C4 | DCLRE1C | B cells_C1 | RABAC1 | Monocyte_C3 | KLF2 |
| CD8+ T cells_C5 | SLC3A2 | B cells_C1 | TXNDC5 | Monocyte_C3 | NR4A1 |
| CD8+ T cells_C5 | CAPZB | B cells_C1 | SLAMF7 | Monocyte_C3 | HSPD1 |
| CD8+ T cells_C5 | TLN1 | B cells_C1 | CRELD2 | Monocyte_C3 | HSPA1B |
| CD8+ T cells_C5 | MYH9 | B cells_C1 | KDELR1 | Monocyte_C3 | JUN |
| CD8+ T cells_C5 | ANXA1 | B cells_C1 | SSR3 | Monocyte_C3 | HSPB1 |
| Treg_C1 | NDUFA11 | B cells_C1 | RPN2 | Monocyte_C3 | HSP90AA1 |
| Treg_C1 | TLN1 | B cells_C1 | P4HB | Monocyte_C3 | HSPH1 |
| Treg_C1 | OST4 | B cells_C1 | FCRL5 | Monocyte_C3 | RHOB |
| Treg_C1 | MYL6 | B cells_C1 | MANEA | Monocyte_C3 | ZFAND2A |
| Treg_C1 | S100A4 | B cells_C1 | TMEM258 | Monocyte_C3 | DNAJB4 |
| Treg_C1 | S100A6 | B cells_C1 | PHPT1 | Monocyte_C3 | UBB |
| Treg_C1 | DNPH1 | B cells_C1 | RRBP1 | Monocyte_C3 | MRPL18 |
| Treg_C1 | ATP5G3 | B cells_C1 | ACADVL | Monocyte_C3 | HSPA6 |
| Treg_C1 | CORO1B | B cells_C1 | TMEM208 | Monocyte_C3 | ID2 |
| Treg_C1 | LGALS1 | B cells_C1 | ANKRD28 | Monocyte_C3 | CCL3 |
| Treg_C1 | MT2A | B cells_C1 | IGLL5 | Monocyte_C3 | CCL4 |
| Treg_C1 | SSR4 | B cells_C1 | LMAN1 | Monocyte_C3 | PDK4 |
| Treg_C2 | FLNA | B cells_C1 | GSTP1 | Monocyte_C3 | NKRF |
| Treg_C2 | NEAT1 | B cells_C1 | SEC61B | Monocyte_C4 | SLC3A2 |
| Treg_C2 | SMCHD1 | B cells_C1 | PPAPDC1B | Monocyte_C4 | CAPZB |
| Treg_C2 | SYNE2 | B cells_C1 | SPCS2 | Monocyte_C4 | PPIF |
| Treg_C2 | KRR1 | B cells_C1 | SIL1 | Monocyte_C4 | TLN1 |
| Treg_C2 | SORL1 | B cells_C1 | TRIB1 | Monocyte_C4 | WARS |
| Treg_C2 | ZC3HAV1 | B cells_C1 | DNAJC1 | B cells_C1 | B4GALT7 |
| Treg_C2 | STAT4 | B cells_C1 | SRM | B cells_C1 | TMED10 |
| Treg_C2 | DHX38 | B cells_C1 | CYBA | B cells_C1 | CTA-292E10.6 |
| Treg_C2 | BPTF | B cells_C1 | SEC24A | B cells_C1 | NDUFB7 |
| Treg_C3 | TLN1 | B cells_C1 | SRPRB | B cells_C1 | TPST2 |
| Treg_C3 | MRPL18 | B cells_C1 | TXNDC15 | B cells_C1 | PDK1 |
| Treg_C4 | ACTN4 | B cells_C1 | NPC2 | B cells_C1 | YIPF3 |
| Treg_C4 | KDM4C | B cells_C1 | MEI1 | B cells_C1 | SAR1B |
| Treg_C4 | BAZ2A | B cells_C1 | ICAM2 | B cells_C1 | UBC |
| Treg_C4 | TMX4 | B cells_C1 | H1FX | B cells_C1 | AQP3 |
| Treg_C4 | CAPZB | B cells_C1 | LMAN2 | B cells_C1 | PIM2 |
| Treg_C4 | NFKBID | B cells_C1 | HSP90B1 | B cells_C1 | MLEC |
| Treg_C4 | TSPYL1 | B cells_C1 | PDIA4 | B cells_C1 | ANAPC11 |
| Treg_C4 | SLC3A2 | B cells_C1 | RPN1 | B cells_C1 | TECR |
| Treg_C4 | APBB1 | B cells_C1 | MAN1A1 | B cells_C1 | HSPA1A |
| Treg_C4 | RP11-727F15.9 | B cells_C1 | SPCS1 | B cells_C1 | ECI1 |
| Treg_C4 | A2M | B cells_C1 | IFI27L1 | B cells_C1 | ITGA6 |
| Treg_C4 | C19orf83 | B cells_C1 | LY96 | B cells_C1 | PDXK |
| Treg_C4 | DNAJB14 | B cells_C1 | PRDM1 | B cells_C1 | NDUFA1 |
| Treg_C4 | HMGB2 | B cells_C1 | CHPF | B cells_C1 | HSPA1B |
| Treg_C4 | UPF2 | B cells_C1 | CD59 | B cells_C1 | TMEM205 |
| Treg_C4 | DDX17 | B cells_C1 | TMED9 | B cells_C1 | CECR1 |
| Treg_C4 | MYH9 | B cells_C1 | PDIA6 | B cells_C1 | LGALS3 |
| Treg_C4 | IQGAP1 | B cells_C1 | KDELR2 | B cells_C1 | IFI27L2 |
| Treg_C4 | CHD2 | B cells_C1 | CD27 | B cells_C1 | ISG20 |
| Treg_C4 | PAQR3 | B cells_C1 | LRPAP1 | B cells_C1 | AURKAIP1 |
| Treg_C4 | VPS13B | B cells_C1 | CLPTM1L | B cells_C1 | ROMO1 |
| Treg_C4 | SLC25A16 | B cells_C1 | SPCS3 | B cells_C1 | NANS |
| Treg_C4 | FBXO7 | B cells_C1 | ERN1 | B cells_C1 | QPCT |
| Treg_C4 | HPS4 | B cells_C1 | FNDC3A | B cells_C1 | LGALSL |
| Treg_C4 | SUMO4 | B cells_C1 | HERPUD1 | B cells_C1 | GLRX |
| Treg_C4 | C2CD5 | B cells_C1 | UBE2J1 | B cells_C1 | TMEM238 |
| Treg_C4 | TANGO2 | B cells_C1 | CD63 | B cells_C1 | PRDX2 |
| Treg_C4 | PTBP2 | B cells_C1 | LAMP2 | B cells_C1 | NDUFB10 |
| Treg_C4 | SIRT7 | B cells_C1 | RP11-731F5.2 | B cells_C1 | PSMB6 |
| Treg_C4 | MT-ND3 | B cells_C1 | CCDC167 | B cells_C1 | UQCRQ |
| Treg_C4 | FCHO1 | B cells_C1 | KRTCAP2 | B cells_C1 | DNAJC3 |
| Treg_C4 | CCDC6 | B cells_C1 | IGJ | B cells_C1 | OSTC |
| Treg_C4 | CNOT6L | B cells_C1 | LMF1 | B cells_C1 | POU2AF1 |
| Treg_C4 | NSMAF | B cells_C1 | TMEM59 | B cells_C1 | REEP5 |
| Treg_C4 | HNRNPA2B1 | B cells_C1 | DPP7 | B cells_C1 | CTSD |
| Treg_C4 | ENTPD4 | B cells_C1 | OS9 | B cells_C1 | VKORC1 |
| Treg_C4 | LETM1 | B cells_C1 | SPATS2 | B cells_C1 | COMMD3 |
| Treg_C4 | SOS2 | B cells_C1 | ERGIC3 | B cells_C1 | CALU |
| Treg_C4 | CMTR2 | B cells_C1 | SEL1L | B cells_C1 | ZBTB8OS |
| Treg_C4 | CHORDC1 | B cells_C1 | HMCES | B cells_C1 | FAM46C |
| Treg_C4 | HLA-F | B cells_C1 | SERF2 | B cells_C1 | MRPL51 |
| Treg_C4 | CRTC2 | B cells_C1 | SEC61A1 | B cells_C1 | WDR45 |
| Treg_C4 | PPIG | B cells_C1 | LINC00152 | B cells_C1 | TMEM147 |
| Treg_C4 | MDFIC | B cells_C1 | PRDX5 | B cells_C1 | SEC62 |
| Treg_C4 | AC137932.1 | B cells_C1 | GAS6 | B cells_C1 | EDF1 |
| Treg_C4 | MR1 | B cells_C1 | MANF | B cells_C1 | CFLAR |
| Treg_C4 | STX7 | B cells_C1 | COPE | B cells_C1 | TMED2 |
| Treg_C4 | CXCR6 | B cells_C1 | SEC61G | B cells_C1 | GNAS |
| Treg_C4 | PHLPP1 | B cells_C1 | TXNDC11 | B cells_C1 | CISD2 |
| Treg_C4 | DHX36 | B cells_C1 | RP11-16E12.2 | B cells_C1 | NDUFA13 |
| Treg_C4 | CD151 | B cells_C1 | SEC14L1 | B cells_C1 | RNF181 |
| Treg_C4 | TAF1B | B cells_C1 | PSAP | B cells_C1 | HSH2D |
| Treg_C4 | RP3-508I15.21 | B cells_C1 | A1BG | B cells_C1 | SSR2 |
| Treg_C4 | YBX3 | B cells_C1 | DNAJB9 | B cells_C1 | NDUFC2 |
| Treg_C4 | CCDC18 | B cells_C1 | DDOST | B cells_C1 | VOPP1 |
| Treg_C4 | TBCK | B cells_C1 | FAM174A | B cells_C1 | SELK |
| Treg_C4 | CHUK | B cells_C1 | AKR1A1 | B cells_C1 | GRN |
| Treg_C4 | SAT1 | B cells_C1 | CCPG1 | B cells_C1 | TYMP |
| Treg_C4 | GPKOW | B cells_C1 | ST6GALNAC4 | B cells_C1 | ATP5D |
| Treg_C4 | EPB41 | B cells_C1 | TRAM1 | B cells_C1 | HSPA5 |
| Treg_C5 | MALAT1 | B cells_C1 | MRPS31 | B cells_C1 | RPS27L |
| Treg_C5 | XIST | B cells_C1 | SELM | B cells_C1 | TCEB2 |
| Treg_C5 | HSPA1B | B cells_C1 | ATOX1 | B cells_C1 | DERL1 |
| Treg_C5 | NEAT1 | B cells_C1 | CNPY2 | B cells_C1 | ATRAID |
| Treg_C5 | TRA2A | B cells_C1 | ALG5 | B cells_C1 | JUN |
| Treg_C5 | TMX3 | B cells_C1 | FIS1 | B cells_C1 | DPEP1 |
| Treg_C5 | CCNT1 | B cells_C1 | EIF2AK4 | B cells_C1 | NDUFA3 |
| B cells_C1 | GYPC | B cells_C1 | UFM1 | B cells_C1 | NDUFB4 |
| B cells_C1 | CANX | B cells_C1 | EAF2 | B cells_C1 | MXD4 |
| B cells_C1 | TIMP2 | B cells_C1 | NDUFB11 | B cells_C1 | IL6ST |
| B cells_C1 | ZBTB38 | B cells_C1 | NDUFS6 | B cells_C1 | IQCG |
| B cells_C1 | COX5A | B cells_C1 | DUSP5 | B cells_C1 | DNAJB1 |
| B cells_C1 | KLF13 | B cells_C1 | ARF4 | B cells_C1 | HIST1H1C |
| B cells_C1 | MZT2B | B cells_C1 | MIR4435-1HG | B cells_C1 | ZFAND2A |
| B cells_C1 | COPZ1 | B cells_C1 | CUTA | B cells_C1 | HSPA1L |
| B cells_C1 | ISCU | B cells_C1 | CD79A | B cells_C1 | SRPR |
| B cells_C1 | SSR1 | B cells_C1 | HSPA6 | B cells_C1 | CST3 |
| B cells_C1 | SMDT1 | B cells_C1 | RPS19BP1 | B cells_C1 | LGALS1 |
| B cells_C1 | RGS1 | B cells_C1 | ALKBH7 | B cells_C1 | ELL2 |
| B cells_C1 | RPL36AL | B cells_C1 | CDK2AP2 | B cells_C1 | IFI6 |
| B cells_C1 | ICAM3 | B cells_C1 | HLA-C | B cells_C1 | CDV3 |
| B cells_C1 | MYL6 | B cells_C1 | BSG | B cells_C1 | PDIA3 |
| B cells_C1 | AP3S1 | B cells_C1 | HSPB1 | B cells_C1 | LMNA |
| B cells_C1 | TMBIM6 | B cells_C1 | BTG2 | B cells_C1 | CCDC144A |
| B cells_C1 | GUK1 | B cells_C1 | HAX1 | B cells_C1 | RRAD |
| B cells_C1 | ZNHIT1 | B cells_C1 | DAD1 | B cells_C1 | PSMB1 |
| B cells_C1 | ATF4 | B cells_C1 | PIM1 | B cells_C1 | TXN |
| B cells_C1 | C19orf60 | B cells_C1 | RER1 | B cells_C1 | COX7A2 |
| B cells_C1 | PRELID1 | B cells_C1 | ZNF706 | B cells_C1 | NEAT1 |
| B cells_C1 | SUB1 | B cells_C1 | FTL | B cells_C1 | RASD1 |
| B cells_C1 | ATP5J | B cells_C1 | ATPIF1 | B cells_C1 | OST4 |
| B cells_C1 | SERP1 | B cells_C1 | CPEB4 | B cells_C1 | BAG3 |
| B cells_C1 | SDF4 | B cells_C1 | SLC3A2 | B cells_C1 | FBXW7 |
| B cells_C1 | ARL6IP4 | B cells_C1 | JTB | B cells_C1 | DNAAF1 |
| B cells_C1 | ATP5A1 | B cells_C1 | MESDC2 | B cells_C1 | RAB30 |
| B cells_C1 | CHMP2A | B cells_C1 | UQCR11.1 | B cells_C1 | UBALD2 |
| B cells_C1 | THAP2 | B cells_C1 | GLA | B cells_C1 | GOLGB1 |
| B cells_C1 | ATF3 | B cells_C1 | RHOB | B cells_C1 | SQSTM1 |
| B cells_C1 | IRF1 | B cells_C2 | NASP | B cells_C3 | CAPZB |
| B cells_C1 | HSP90AA1 | B cells_C2 | ITSN2 | B cells_C3 | CD52 |
| B cells_C1 | DNAJB4 | B cells_C2 | REL | B cells_C3 | MIR142 |
| B cells_C1 | XIST | B cells_C2 | CD55 | B cells_C4 | TLN1 |
| B cells_C1 | TIMP1 | B cells_C2 | KDM6B | B cells_C5 | MYH9 |
| B cells_C1 | AC006129.4 | B cells_C2 | TMEM2 | B cells_C5 | IQGAP1 |
| B cells_C1 | PPP1R15A | B cells_C2 | SKIL | B cells_C5 | IRS2 |
| B cells_C1 | OTUD1 | B cells_C2 | RUNX3 | B cells_C5 | LBH |
| B cells_C1 | KIAA0125 | B cells_C2 | LDLRAD4 | B cells_C5 | SLC2A3 |
| B cells_C2 | FLNA | B cells_C2 | RANBP2 | B cells_C5 | TNKS2 |
| B cells_C2 | GRASP | B cells_C2 | HNRNPU-AS1 | B cells_C5 | GPRIN3 |
